# Homoeolog-specific retention and use in allotetraploid *Arabidopsis suecica *depends on parent of origin and network partners

**DOI:** 10.1186/gb-2010-11-12-r125

**Published:** 2010-12-23

**Authors:** Peter L Chang, Brian P Dilkes, Michelle McMahon, Luca Comai, Sergey V Nuzhdin

**Affiliations:** 1 Molecular and Computational Biology, University of Southern California, 1050 Childs Way, RRI 201, Los Angeles, CA 90089-2910, USA; 2 Genome Center and Department of Plant Biology, University of California at Davis, 451 Health Services Drive, Davis, CA 95616, USA; 3 Current address: Department of Horticulture and Landscape Architecture, Purdue University, 625 Agriculture Mall Drive, West Lafayette, IN 47907-2010, USA; 4 School of Plant Sciences, University of Arizona, 1140 E. South Campus Drive, Forbes Building, Room 303, Tucson, AZ 85721-0036, USA

## Abstract

**Background:**

Allotetraploids carry pairs of diverged homoeologs for most genes. With the genome doubled in size, the number of putative interactions is enormous. This poses challenges on how to coordinate the two disparate genomes, and creates opportunities by enhancing the phenotypic variation. New combinations of alleles co-adapt and respond to new environmental pressures. Three stages of the allopolyploidization process - parental species divergence, hybridization, and genome duplication - have been well analyzed. The last stage of evolutionary adjustments remains mysterious.

**Results:**

Homoeolog-specific retention and use were analyzed in *Arabidopsis suecica *(*As*), a species derived from *A. thaliana *(*At*) and *A. arenosa *(*Aa*) in a single event 12,000 to 300,000 years ago. We used 405,466 diagnostic features on tiling microarrays to recognize *At *and *Aa *contributions to the *As *genome and transcriptome: 324 genes lacked *Aa *contributions and 614 genes lacked *At *contributions within *As*. In leaf tissues, 3,458 genes preferentially expressed *At *homoeologs while 4,150 favored *Aa *homoeologs. These patterns were validated with resequencing. Genes with preferential use of *Aa *homoeologs were enriched for expression functions, consistent with the dominance of *Aa *transcription. Heterologous networks - mixed from *At *and *Aa *transcripts - were underrepresented.

**Conclusions:**

Thousands of deleted and silenced homoeologs in the genome of *As *were identified. Since heterologous networks may be compromised by interspecies incompatibilities, these networks evolve co-biases, expressing either only *Aa *or only *At *homoeologs. This progressive change towards predominantly pure parental networks might contribute to phenotypic variability and plasticity, and enable the species to exploit a larger range of environments.

## Background

An allotetraploid is formed when diploids from two different species, which may have diverged for millions of years, hybridize. The resulting plant, if viable, might have a competitive edge, such as broader ecological tolerance compared to its parents [1-3]. The evolutionary importance of polyploidy, of which allotetraploidy is a common form, is reflected in its prevalence in flowering plants [4]: ancient polyploidy is apparent in all plant genomes sequenced to date and is estimated to have been involved in 15% of all plant speciation events [5]. Furthermore, most cultivated crops have undergone polyploidization during their ancestry [5,6]. Why are polyploids so evolutionarily, ecologically, and agriculturally successful? To answer this question, one has to consider the evolutionary and genetic processes acting at different stages of polyploidization.

Allopolyploidization can be characterized by four distinct stages. Stage 1 is the divergence between parental species, with both species adapting to specific environments and adopting their own mating strategies and reproductive schedules. Directional selection can contribute to the fixation of species-specific beneficial mutations in coding and regulatory regions [7,8], while slightly deleterious mutations are introduced due to drift. In stages 2 and 3, the diverged species hybridize and increase ploidy, with the two events sometimes reversed in order [9]. This change in ploidy enables the correct pairing at meiosis. Hybridization frequently results in phenotypic instability, widespread genomic rearrangements, epigenetic silencing, and unusual splicing [3,10-25]. Newly created polyploids often experience rapid intragenomic adjustments. Stages 2 and 3 are well-studied with artificial polyploids constructed in the laboratory [10,12-17,19,22-24] or spontaneously arising in nature [14,26].

Stage 4 is the long term evolution of homoeologous genes (that is, homologous genes from two parents joined into one polyploid genome and stably inherited). This stage occurs much slower on the evolutionary time-scale and has received considerably less attention, perhaps due to several technical limitations. Sequence analyses have historically required extensive cloning and bioinformatics. Microarrays have had to be specifically designed to distinguish between homoeologs and orthologs. Interesting patterns have been reported, but typically for a few genes [14,27-29]. Notably, the retention and expression of homoeologs is frequently biased towards one parental species. These patterns were reported on a large scale for approximately 1,400 out of 42,000 genes in cotton [30-32], and for dozens in *Tragopogon *[33]. Recent studies have also discovered abundant genetic variation among independently originated or evolved accessions of *Tragopogon *[34-36]. What molecular evolutionary processes account for this variation among accessions? How does intraspecific variation in polyploid genomes contribute to phenotypic variation? These questions remain wide open.

Here, we focus on *Arabidopsis suecica *(*As*), a highly selfing species [37] found mainly in central Sweden and southern Finland [38]. *As *originated 12,000 to 300,000 years ago (KYA) from a cross between a largely homozygous ovule-parent *Arabidopsis thaliana *(*At*, 2n10) and a pollen-parent *Arabidopsis arenosa *(*Aa*, 2n = 16) [39-41]. A single origin of *As *(2n = 26) has been established with mitochondrial, chloroplast, and nuclear DNA [39-41]. *As *originated south of the ice cover and spread north when the ice retreated 10,000 years ago [39]. *At *is an annual, weedy, and mostly autogamous species native to Europe and central Asia but naturalized worldwide [42]. It has undergone at least two rounds of ancient polyploidization [26] and is annotated with 39 thousand genes. *Aa *is a self-incompatible member of the *Arabidopsis *genus, carrying the highest level of genetic diversity among the species group [43]. *At *and *Aa *diverged approximately 5 million years ago [44].

One can generate an artificial F_1 _allotetraploid (*F*_*1*_*As*) in the lab by performing a cross between a tetraploid *At *ovule-parent and a tetraploid *Aa *pollen donor. The resulting primary species hybrid contains two genomes from *At *and two from *Aa*. We can use this as an estimate, as the exact haplotypes that contributed to the initial hybridization event are not available, of the genomic composition and homoeolog-specific expression at the time of allopolyploid speciation [24,45,46]. Taking these patterns as reflective of the *As *ancestral state, we observed how evolution has shaped the *As *genome. As *At *is a selfer and *Aa *an outcrosser, *At*-originated homoeologs might have possessed more deleterious mutations due to Hill-Robertson interference [47]. Are *Aa*-originated homoeologs more commonly retained? *At *and *Aa *evolved orthologous networks in which genes were finely tuned to coordinate, separately within each species. Interference of *At *and *Aa *homoeologs may cause mis-regulation within mixed *As *networks. This is akin to Dobzhansky-Muller incompatibilities [48]. Do heterologous networks evolve to restore their original orthologous-like compositions? Here, we address these and other questions.

## Results

For every gene in *As*, we set to determine whether both *At *and *Aa *homoeologs are present in the genome and whether they are expressed evenly or in homoeolog-specific fashion [49]. With the genome-wide *Arabidopsis *tiling microarray, we scanned the genomes of *At*, *Aa*, *As*, and *F*_*1*_*As*. We analyzed the transcriptome of *As *with tiling arrays and validated results with Illumina resequencing. We assembled a statistical pipeline to identify *At *and *Aa *homoeolog-originated signals, and to estimate their contribution to the *As *populations of DNA and RNA.

### Comparison of probe hybridization between parental species, and between *As *and *F*_*1*_*As*

**Figure 1:**
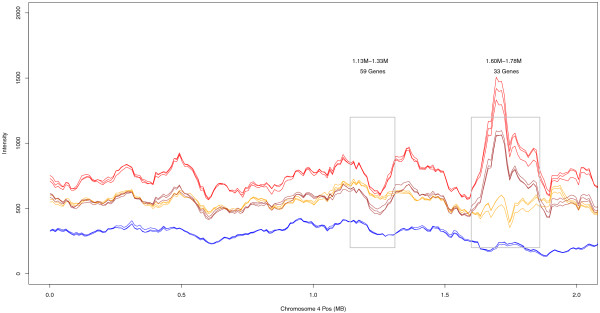
**Chromosomal distribution of probe intensities**. The 100-kb sliding window averages for *At *(red), *Aa *(blue), *As *(gold), and *F*_*1*_*As *(brown) on chromosome 4. Chromosome positions and gene annotations correspond to the *At *genome. Gray boxes indicate clusters containing at least 30 genes with a strong unidirectional bias, where at least 27 genes have the same bias, and significant for at least 9 genes. A list of clusters can be found in Table 1. Genes within these clusters can be found in Additional file 2.

**Figure 2:**
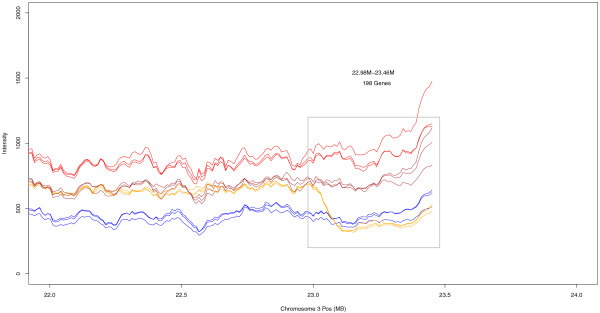
**Chromosome distribution of probe intensities**. The 100-kb sliding window averages for *At *(red), *Aa *(blue), *As *(gold), and *F*_*1*_*As *(brown) on chromosome 3.

The *Arabidopsis *array features 3.2 million 25-base-long probes tiled throughout the complete genome at a 35-base distance. As these features are homologous to the *At *reference, they should, on average, exhibit a lower hybridization with *Aa *DNA. Probe intensities confirm this expectation. Two typical examples are shown for chromosomes 3 and 4 (Figures 1 and 2; see Additional files 1, 2, 3,4, 5 and 6 for other examples). *F*_*1*_*As *signals are a sharp intermediate between *At *and *Aa*. *As *shows remarkable correspondence with *F*_*1*_*As*, with the exception of several extended regions. We hypothesize that these regions correspond to historic losses of homoeologous chromosomal regions in *As*.

We mapped features onto the genes and compared intensities between *As *and *F*_*1*_*As*; 6,790 genes exhibited differential hybridization (Wilcoxon ranked sum test, false discovery rate (FDR) <0.05). To identify large putative alterations, we scanned for clusters containing at least 30 genes with a strong unidirectional bias (at least 27 with the same bias, significant for at least 9 genes). We identified 39 clusters, encompassing 1,643 genes (Table 1). Some clusters were due to differential abundance of transposable-element-like sequences. Chr1 13.66 M, Chr1 14.00 M, Chr3 12.44 M, Chr3 13.36 M, and Chr5 11.06 M mainly consisted of *copia*-like, *gypsy*-like, or *CACTA*-like retrotransposons. Other regions - for instance, on Chr1 0.29 M, Chr3 0.30 M, Chr3 5.58 M, Chr3 21.60 M, and Chr3 22.98 M - appeared free from this problem (Additional file 2 includes detailed information). Interestingly, the region 1.60 M-1.78 M on chromosome 4 (Figure 1) is coincident with the heterochromatic knob known to be hypervariable in *At *[50]. The 22.98 M-23.46 M region of chromosome 3 (Figure 2) looked like an *At*-homoeolog deletion. These results show that tiling arrays can be a useful tool for detecting copy number variation [51] and large-scale alterations in the *As *genome. As these analyses are based on non-normalized signals (between species), they are likely error-prone for individual genes.

**Table 1:** Regions of putative alterations in *Arabidopsis suecica*

Chromosome	Region	Number of genes	Percent with differential hybridization	Percent TEs	Number of probes	Higher hybridization in?
AT1	0.29 M-0.39 M	38	44.7	0	2,537	*F*_*1*_*As*
	0.82 M-0.91 M	32	28.1	3.1	2,266	*F*_*1*_*As*
	3.16 M-3.29 M	43	37.2	0	3,175	*As*
	8.40 M-8.49 M	37	29.7	2.7	1,991	*F*_*1*_*As*
	13.66 M-13.86 M	43	58.1	51.2	3,547	*F*_*1*_*As*
	14.00 M-14.39 M	70	42.9	51.4	5,998	*F*_*1*_*As*
	29.97 M-30.07 M	40	32.5	0	2,536	*F*_*1*_*As*
AT2	1.96 M-2.03 M	34	32.4	8.8	1,377	*As*
	4.57 M-4.69 M	30	30.0	36.7	2,302	*F*_*1*_*As*
	6.50 M-6.67 M	43	27.9	16.3	3,214	*As*
	10.88 M-11.01 M	38	26.3	0	3,182	*As*
	14.74 M-14.84 M	37	27.0	0	2,440	*F*_*1*_*As*
	19.60 M-19.68 M	36	38.9	0	2,065	*F*_*1*_*As*
AT3	0.30 M-0.36 M	33	42.4	0	1,568	*F*_*1*_*As*
	5.58 M-5.68 M	32	46.9	0	2,299	*As*
	7.30 M-7.38 M	31	32.3	16.1	1,822	*F*_*1*_*As*
	12.44 M-12.61 M	36	27.8	61.1	3,055	*F*_*1*_*As*
	13.36 M-13.50 M	34	55.9	50.0	2,431	*As*
	14.55 M-14.70 M	39	38.5	33.3	2,904	*As*
	20.25 M-20.34 M	31	32.3	3.2	2,165	*F*_*1*_*As*
	20.93 M-21.00 M	30	30.0	0	1,881	*F*_*1*_*As*
	21.30 M-21.43 M	44	34.1	2.3	3,227	*F*_*1*_*As*
	21.60 M-21.73 M	45	44.4	0	3,217	*F*_*1*_*As*
	22.11 M-22.22 M	37	29.7	0	2,520	*F*_*1*_*As*
	22.98 M-23.46 M	198	79.8	2.0	12,309	*F*_*1*_*As*
AT4	1.13 M-1.33 M	59	28.8	1.7	4,967	*As*
	1.60 M-1.78 M	33	57.6	39.4	2,762	*F*_*1*_*As*
	7.59 M-7.68 M	34	29.4	2.9	2,052	*As*
	7.67 M-7.82 M	47	23.4	21.3	3,232	*As*
	16.89 M-16.96 M	32	34.4	0	1,797	*As*
	17.86 M-17.95 M	39	38.5	0	2,000	*F*_*1*_*As*
AT5	9.92 M-10.11 M	44	43.2	22.7	4,269	*As*
	11.06 M-11.27 M	42	45.2	59.5	2,948	*F*_*1*_*As*
	13.76 M-13.89 M	38	36.8	18.4	2,785	*As*
	18.49 M-18.61 M	33	30.3	0	2,882	*As*
	20.53 M-20.70 M	34	29.4	2.9	2,621	*As*
	23.48 M-23.56 M	33	30.3	0	1,991	*F*_*1*_*As*
	26.41 M-6.47 M	34	29.4	0	1,453	*F*_*1*_*As*
ATM	0.02 M-.24 M	30	50.0	0	1,447	*F*_*1*_*As*

*As*, *Arabidopsis suecica*; *F1As*, *F1 artificial allotetraploid*.

### Homoeolog-specific retention

**Figure 3:**
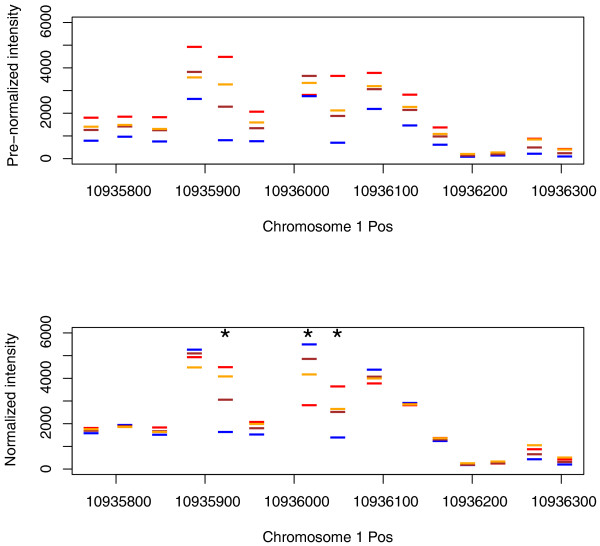
**Probe intensities before and after normalization**. Probe intensities for every gene were normalized to identical levels in all arrays. A *t*-test between *At *(red) and *Aa *(blue) replicates identified diagnostic features (shown with asterisks) that were used to identify homoeolog-specific hybridization. *F*_*1*_*As *(brown) is shown as a null reference for which to compare *As *(gold).

To analyze the homoeolog-specific retention and expression of individual genes, we focused on 1,393,557 probes mapping to coding regions using Bowtie [52]. Since *Aa *and *At *sequences differ at 1 out of 20 bases, some 25-base oligonucleotides designed for *At *are a perfect match for *Aa *sequences. Whenever orthologous *Aa *sequences mis-match to the *At *chip, this hybridization is weakened (hereafter termed 'diagnostic features' (DFs)). Separately for every gene, we identified a scaling factor based on probes with similar signatures of hybridization to normalize intensities between species. We then identified homoeolog-specific DFs and only retained those (405,466) robust over replicates (Figure 3). We could only follow 24,344 genes as the fastest-evolving genes have too many DFs for normalization (Additional file 3).

**Figure 4:**
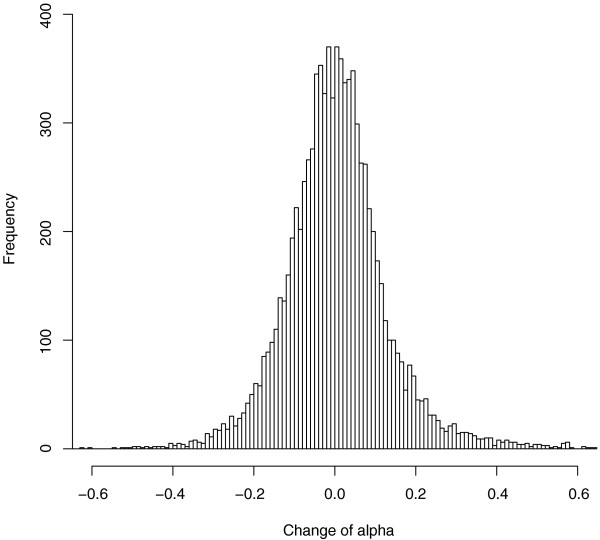
**Histogram distribution of homoeolog bias Δα**. Δα is shown for the genome of *As*, using *F*_*1*_*As *as a null reference. Distribution is nearly symmetrical and centered at 0.004.

We tested for deviations from an equal representation of the two homoeologs in the *As *genome [12,16,53]. As a reference point, we used the *F*_*1*_*As *DNA in which homoeologs are present at equal doses (Figure 1). For each gene within the regions of putative alterations, we tested for changes in α between *As *and *F*_*1*_*As*, where α represents the relative contribution of *Aa *DF hybridization strengths in a hybrid genome. There was an upward shift in α in *As *compared to *F*_*1*_*As *(one-sided paired *t*-test, *P *< 2e-17), suggesting a preferential retention of homoeologs derived from the *Aa *parent (Figure 4). Supporting this, more genes were called *Aa*-like (614) than *At*-like (324). This bias is significant, although moderate compared to earlier studies [30-32,34-36]. This might reflect a limited power of microarrays. For instance, we analyzed 30 genes encoded by the mitochondria organelle known to be *At*-derived. Only one plastid-encoded gene had enough DFs to be unambiguously classified, and was biased towards maternal *At*, as expected.

### Use of *At *and *Aa *homoeologs in *As *transcriptome

**Figure 5:**
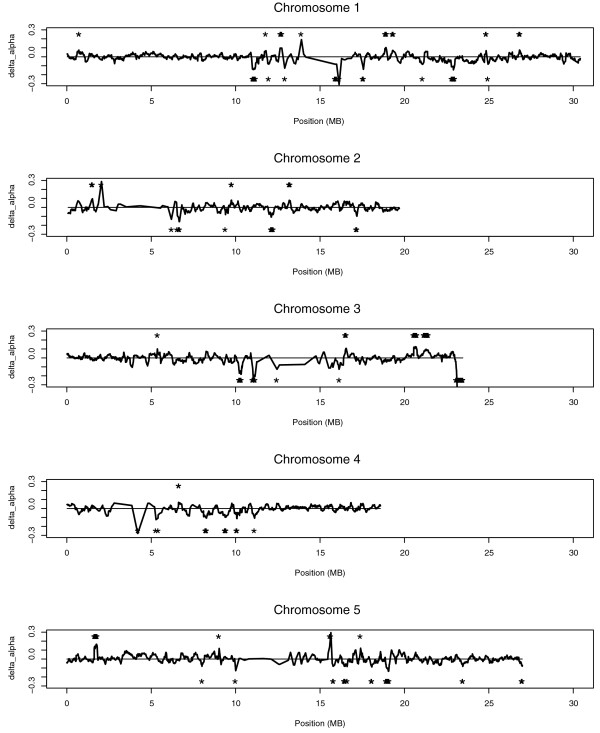
**Chromosomal distribution of clusters of biased homoeolog transcripts**. Lines above the center indicate clusters of *At*-like genes, and those below indicate of *Aa*-like genes. Asterisks depict significance using a genome-wide permutation test. Presence of another asterisk indicates a nearby region that is also clustered with *At*- or *Aa*-enriched transcription.

To identify homoeologous transcripts in *As*, we extracted RNA from leaf tissues and processed microarrays with the SNP-detection protocols similar to above. More than 49% of genes were called expressed, and 7,608 exhibited homoeolog-specific expression, with 3,458 and 4,150 exhibiting *At*-enriched and *Aa*-enriched DFs, respectively. Overall, we conclude that, over the 12,000 to 300,000 years, *As *has accumulated more deletions of *At*-originated homoeologs and uses the remaining *At*-originated homoeologs somewhat less (Table 2). Genes physically clustered together might co-express and co-evolve in transcript levels, as previously observed in flies [54]. To test whether biases in homoeolog-specific expression were concordant between nearby genes, we calculated running averages of Δα along chromosomes (Figure 5), and found regions with clusters of *At*-enriched and *Aa*-enriched transcription.

**Table 2:** Homoeolog-specific retention and use in *Arabidopsis suecica*

Classification	*As *genome	*As *transcriptome
*At*-like	324	3,458
*Aa*-like	614	4,150

*Aa*, *Arabidopsis arenosa*; *As*, *Arabidopsis suecica*; *At*, *Arabidopsis thaliana*.

**Figure 6:**
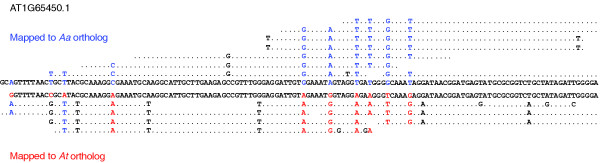
**Sequenced read alignments to *At *and *Aa *orthologs**. Orthologous *At *and *Aa *sequences shown at center contain diagnostic SNPs in red and blue, respectively, that can be used to align and cluster Illumina reads.

**Figure 7:**
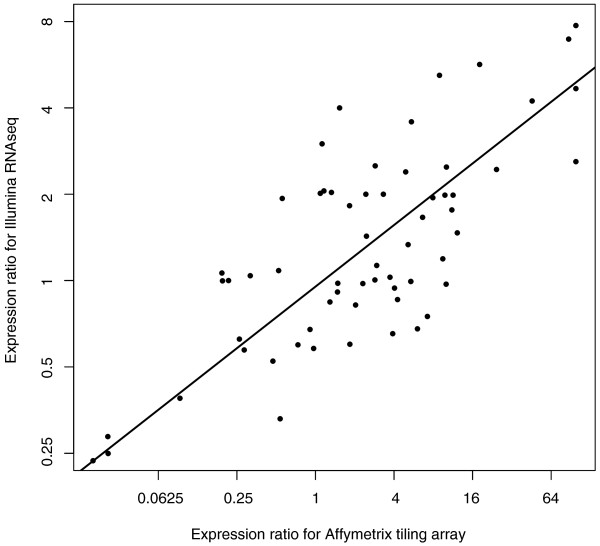
**Concordance between homoeolog-specific expression estimated from *At *tiling microarray (X-axis) and Illumina resequencing (Y-axis)**. R^2 ^= 0.646, *P *< 5e-07.

To validate the tiling array-based procedures above, we prepared Illumina libraries and performed RNA-sequencing of the *As *transcriptome. The *Aa *genome is not yet assembled, but we identified 52 *Aa *genes from GenBank and acquired an additional 50 genes from the UC Genome Center. We identified the orthologous *At *genes for these *Aa *genes and mapped the Illumina reads to both homologs. Nine genes did not contain any reads that were mapped to either homolog. For 14 genes, reads only mapped to either the *Aa *or the *At *reference. For the remaining genes, reads were aligned to both homologs and clustered as either derived from *At *or *Aa *(Figure 6). We consider the number of uniquely mapped reads as a measure of homoeolog-specific expression. A strong correlation in *Aa*:*At *expression ratio between tiling arrays and the RNA-seq (R^2 ^= 0.646, *P *< 5e-07) proves that both approaches work. This concordance is very satisfactory (Figure 7) given that RNA samples were extracted from independently grown plants, and that microarray estimates are frequently noisy.

### Network analyses of homoeolog-specific genes

The summary of the Gene Ontology analysis of genes exhibiting homoeolog-specific retention and expression is shown in Tables 3 and 4. The categories 'cell communication' and 'signal transduction' were underrepresented, while 'DNA repair' and 'response to DNA damage stimulus' were overrepresented. *Aa*-enriched transcripts were overrepresented in the 'gene expression' category, including subprocesses involved in transcription, translation, RNA processing and gene silencing by miRNA.

**Table 3:** Gene Ontology annotation for homoeolog-biased genes in the *Arabidopsis suecica *genome, overrepresented unless stated

Classification	Biological process	*P*-value
*At*-like	Sulfur amino acid metabolic process	0.00078
	Response to fungus	0.0054
	Heat acclimation	0.0054
	Aspartate family amino acid metabolic process	0.012
	mRNA metabolic process	0.012
	Riboflavin biosynthetic process	0.013
	Membrane lipid metabolic process	0.013
	Cellular sodium ion homeostasis	0.013
	Cellular calcium ion homeostasis	0.021
	Aspartate family amino acid metabolic process	0.024
	Purine ribonucleoside monophosphate metabolic process	0.035
	Cellular potassium ion homeostasis	0.036
*Aa*-like	Protein amino acid glycosylation	0.021
	Defense response, underrepresented	0.029
	DNA repair	0.024
	Response to DNA damage stimulus	0.024
	RNA metabolic process	0.028
	Cell communication, underrepresented	0.031
	Signal transduction, underrepresented	0.033
	Hormone transport	0.044
	Microtubule cytoskeleton organization	0.044

**Table 4:** Gene Ontology annotations for homoeolog-biased use (expression) in *Arabidopsis suecica *transcriptome, overrepresented unless stated

Classification	Biological process	*P*-value
*At*-like	One-carbon metabolic process	6.1e-05
	Intracellular protein transport	0.00012
	Macromolecule localization	0.00012
	Microtubule-based movement	0.00045
	Cytoskeleton-dependent intracellular transport	0.00045
	Protein complex assembly	0.0030
	Cellular component organization	0.0039
	Cytoskeleton organization and biogenesis	0.0039
	Photorespiration	0.0053
	Seryl-tRNA aminoacylation	0.0069
	Aspartate family amino acid metabolic process	0.0071
	mRNA metabolic process	0.011
	Response to drug, underrepresented	0.020
	Drug transport, underrepresented	0.020
	Pyrimidine base metabolic process	0.024
	Phosphate transport	0.024
	Inflammatory response	0.024
*Aa*-like	Oxidative phosphorylation	0.0013
	ATP synthesis coupled electron transport	0.0024
	Programmed cell death	0.0028
	Cell development	0.0043
	Glycerol metabolic process	0.0058
	Alcohol metabolic process	0.0058
	Hormone metabolic process	0.0058
	Phagocytosis	0.0081
	Endocytosis	0.0081
	Hormone catabolic process	0.012
	Photomorphogenesis	0.014
	tRNA metabolic process, underrepresented	0.017
	Transcription	0.023
	Nuclear transport	0.031
	Regulation of cell cycle	0.034
	RNA polyadenylation	0.034

**Figure 8:**
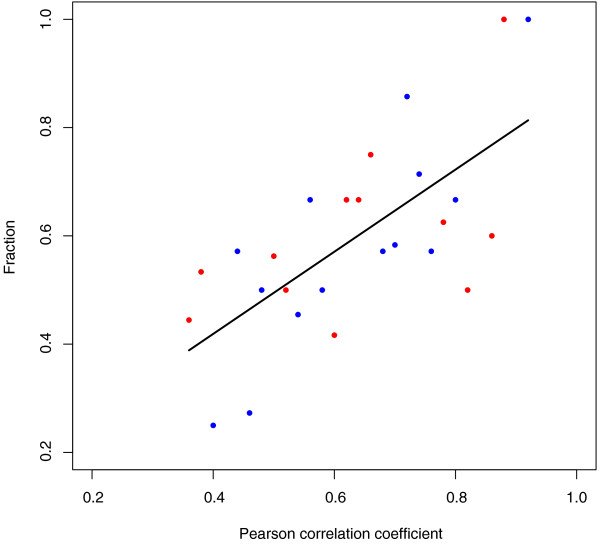
**Fraction of gene pairs co-biased as either *At *or *Aa *for bins of different connectivity**. R-squared = 0.47, *P *< 0.0001. Red dots represent bins with higher fraction of *At *co-biased genes within bin. Blue dots represent bins with higher fraction of *Aa *co-biased genes within bin.

Lastly, we considered homoeolog-specific expression in the context of *At *transcriptional networks [55]. Of the 7,608 genes, connectedness estimates were available for 6,941 gene pairs. We tested whether bins of higher-connected gene pairs exhibited higher concordance of homoeolog-specific expression (Figure 8). The fraction of concordant pairs was approximately 0.4 in low-connectedness bins, but increased to 0.8 for the high-connected gene pairs (R^2 ^= 0.47, *P *< 0.0001). We also partitioned networks with homoeolog-specific expressions of at least two genes as co-biased for *Aa *(325), co-biased for *At *(219), or with mixed biases (302) (Table 5). The latter 'mixed' group was significantly underrepresented in comparison with random expectation (χ^2 ^test, *P *< 6e-08).

**Table 5:** Co-biased pairs of *Arabidopsis suecica *homoeologs in *Arabidopsis thalianat*-identified gene networks

Classification	Co-biased as *At*	Biased as *At *and *Aa*	Co-biased as *Aa*
Occurrence	219	302	325
Expected	173.1	419.2	253.7

χ^2 ^test, *P *< 6e-08.

## Discussion

In allopolyploid speciation, two genomes that have experienced long independent evolution are combined. Their genomes were shaped in different ways in response to the extrinsic environmental and intrinsic lifestyle pressures. We focused on *As*, a species that evolved 12 to 300 KYA from a single hybrid individual formed from an ovule of *At *and a pollen of *Aa*. Orthologous genes of *At *and *Aa *have average sequence divergence of 5% [43], exhibit differences in tissue-specific expression [10,24], and are located on five versus eight chromosomes. The allotetraploid hybrid initially had low fertility, if one can conclude this from the performance of artificial hybrids in the lab. This fertility can be restored through the complex interplay of genetic and epigenetic processes [22]. Several groups have been fascinated with this rapid but complex process [10,22,24,45,46,53,56-59]. We focus on the subsequent longer-term molecular evolution, by comparing an evolved natural *As *with an 'unevolved' *F*_*1*_*As *hybrid.

### The summary of *F*_*1*_*As *unevolved patterns

*F*_*1*_*As *and its following generations are a model for whole-genome rearrangements and gene expression. Approximately one of ten cDNA amplified fragment length polymorphism (AFLP) bands displayed patterns that were non-additive between *F*_*1*_*As *and its parental species [16]. One percent of bands were not detected in the parental species altogether [24]. For AFLP fragments observed in the parents, homoeolog silencing was nearly symmetrical: 4% of *At *versus 5% of *Aa*. These patterns varied among tissues in a seemingly stochastic way. There was also some variation among accessions. In addition to AFLPs, Wang *et al*. [53] used spotted 70-mer oligonucleotide arrays to compare gene expression between *At*, *Aa*, and *F*_*1*_*As*. More than 15% of transcripts had different levels between parental species. In *F*_*1*_*As*, 5% of genes deviated in expression level from the additive mid-parent expectation, with the majority being repressed. Interestingly, 94% of these genes were more strongly expressed in the *At *parent, with their levels of expression in *F*_*1*_*As *resembling *Aa *[56,57]. In conclusion, the levels of gene expression in *F*_*1*_*As *more frequently resemble those in *Aa*, although homoeologs seem to have been used symmetrically and sometimes randomly. *Aa*-specific phenotypes, such as flower morphology, plant stature and long lifespan, are dominant in *F*_*1*_*As *(likewise, *Arabidopsis lyrata *phenotypes are dominant in *thaliana*-*lyrata *hybrids [56,59]). These results were confirmed and further detailed in very recent investigations [24,45,46].

### Evolved *As *patterns

We found that in *As*, *Aa *homoeologs are more frequently retained and more actively transcribed than their *At *counterparts. We hypothesize that these *Aa*-favoring biases are not random, but rather represent a signature of an evolutionary process. To explain these patterns, we propose a concept of 'homoeolog competition.' Genes are subject to detrimental mutations at approximately constant rates [47]. Purifying selection removes these mutations with varying efficiencies depending on the gene redundancy, dominance, and other characteristics [6,21,60,61]. As some *F*_*1*_*As *homoeologs are functionally redundant, they should be progressively lost to mutations and deletions. From the initial pool of homoeologs, natural selection would preferentially maintain those with a higher contribution to fitness. In this sense, homoeologs 'compete'. Despite stoichiometric constraints to maintain stable ratios of dosage among genes [62], there is a well-documented shrinkage of polyploid genomes over time [6,9,12,15,18,21,25,26], as few genes are haploinsufficient [60].

Why would *At*-originated homoeologs be less valuable? Our first hypothesis is inspired by Hill and Robertson [60]. Selfing organisms, such as *At*, are less capable of purging mildly deleterious mutations. This is because of severely reduced recombination in comparison to outcrossers, such as *Aa *[61,63,64]. This may seem paradoxical, as *At *maintains much less variation than *Aa *[43], which one might interpret as mutations in *Aa*. When selfing evolves, segregating mutations are quickly purged, as they exhibit their deleterious nature in autozygous individuals. In the short term, selfers are in fact better off [61]. With time, however, Mullers' ratchet kicks in one slightly deleterious mutation after another, resulting in low standing variation but inferior functionality [47]. Selfing is typical of terminal branches on phylogenetic trees, interpreted as being an evolutionary dead-end [64,65]. Thus, *Aa *homoeologs may contribute more to the fitness of an *F*_*1*_*As*, as they originate from an outcrossing species. In the future, we will test this hypothesis by population 'allele-specific' resequencing and applying molecular evolution tests to homoeologs separately.

Our second hypothesis involves historical factors. Suppose the southern-adapted *At *accession hybridized with the northern-adapted *Aa *accession, and that the emerging *As *accession spent most of the 12,000 to 300,000 years in the northern environment [37,39]. *Aa*-originated homoeologs would be a better fit for the environment, would be more frequently retained, and would evolve to be preferentially used [66]. To test this hypothesis, one must sample *As *accessions from multiple locations, resequence their genomes and transcriptomes and identify environment-specific molecular evolution since the unique *As *speciation event. Our model assumes a large standing variation in the genome and transcriptome, which has been well-documented in *Tragopogon *[35,36]. A more direct, rather than biogeographic-type, evidence might be obtained with *Gossypium *[14]. This species displays a similar strengthening of parentally skewed expression when natural allotetraploids are compared with F_1 _allotetraploid controls.

Thirdly, recall that the *Aa *transcription machinery is preferentially expressed in *F*_*1*_*As *[53]. Homoeologs pre-adapted to function under *Aa *transcriptional control will then be selected for, reinforcing this initial pattern. Homoeolog-specific methylation might be at the heart of these processes [45,46]. Indirectly supporting this idea, *Aa*-like genes exhibited enrichment in the 'gene expression' category (with subprocesses: transcription, translation, RNA processing, and gene silencing by miRNA). Recent reports in *Arabidopsis *and *Brassica *allopolyploids indicate a high proportion of nonadditive expression for genes within these categories as well [53,67,68]. Similar results have also been shown in *Senecio *[69,70].

### Resolving incompatibilities in allotetraploid networks

Imagine ancestral genes *A1 *and *A2 *that formed a functional dimer in the common ancestor of *Aa *and *At *5 million years ago. These genes evolved into *At1 *and *At2 *orthologs in the *At *lineage, and into *Aa1 *and *Aa2 *orthologs in the *Aa *lineage. Within these lineages, *At1 *and *At2 *have been selected for the ability to form a dimer. Likewise, co-evolution has been taking place between *Aa1 *and *Aa2 *proteins [48]. In *F*_*1*_*As*, along with the parental dimers *At1*-*At2 *and *Aa1*-*Aa2*, there will also be heterologous *At1*-*Aa2 *and *Aa1*-*At2 *dimers. Are these dimers likely to be functional [48]? Dobzhansky and Muller hypothesized that some would not be [71]. Strongly decreased fitness of *At *× *Aa *F_1 _and F_2 _seeds, and meiotic disruptions in F_1_'s, attest to the presence of intrinsic incompatibilities contributing to the reproductive isolation of these two species, and some genes involved have been characterized [61,62].

An allotetraploid might walk an evolutionary path to fitness restoration by preferentially co-expressing only one parental set of interacting homoeologs, with mixed networks being less common. The data confirmed our expectation that homoeologous networks in fact evolved towards pure *Aa *or *At *profiles. This type of 'D-M homoeolog conflict resolution' should be typical for polyploid ancestors and might potentially contribute to the fractionated genomes we observe today [9,72]. As we now know the identity of networks having evolved to a 'pure' parental type, our strong prediction is that the experimenter-induced heterologous state in these networks shall result in detectable reproductive losses.

## Conclusions

When an allotetraploid is formed, the functions of homoeologs are partially redundant, and the genome is set for gene silencing and deletion. Thousands of genes affected by these processes in *As *were identified with tiling arrays and resequencing. These new computational approaches enable the use of widely available and economical tiling microarrays for the whole-genome analyses of species closely related to the sequenced references. In the *As *allotetraploid, more *At*-originated homoeologs are lost and silenced than *Aa*-originated homoeologs. We hypothesize that these *Aa*-favoring biases are not random, but rather represent a signature of an evolutionary process. Whenever more than one gene experiences silencing within a network, the homoeolog bias of the first event influences the likewise bias for the subsequent silencing; networks evolve towards their ancestral types. The mosaics of predominantly pure-parental networks in allotetraploids might contribute to phenotypic variability and plasticity, and enable the species to exploit a larger range of environments.

## Materials and methods

### Plant material, DNA and RNA extractions

Affymetrix GeneChip^® ^*Arabidopsis *Tiling 1.0R Arrays were hybridized with samples from four different sources. Genomic DNA was obtained from tetraploid *At *accession Ler [73], tetraploid *Aa *accession Care-1 [58], allotetraploid *As *accession Sue-1 [73], and an *F*_*1*_*As *produced by crossing the tetraploids *At *and *Aa *as maternal and paternal parents, respectively [58]. cDNA was prepared from *As *leaf samples. All genomic DNA and cDNA samples were hybridized in three biological replicates using standard protocols.

### Sample Illumina library preparation

RNA purification, cDNA synthesis and Illumina library construction was performed using the protocols of Mortazavi *et al*. [74] with the following modifications. Total RNA, mRNA, and DNA were quantified using a Qubit fluorometer (Invitrogen, Carlsbad, CA, USA). mRNA fragmentation was performed using Fragmentation Reagent (Ambion, Austin, TX, USA) and subsequently cleaned through an RNA cleanup kit (Zymo Research, Irvine, CA, USA). Additional DNA and gel purification steps were conducted using Clean and Concentrator kits (Zymo Research). Illumina sequences are available for download at the NCBI Short Read Archive under the accession SRA025958.

### Microarray preprocessing and normalization

The *Arabidopsis *Tiling Microarray is composed of over 3.2 million probe pairs tiled throughout the complete *At *genome. Probes are tiled at an average of 35 base pairs. Affymetrix CEL files are available for download from the public repository ArrayExpress under the accessions E-MEXP-2968 and E-MEXP-2969. To ensure that arrays within genotypes are comparable to each other, Robust Multiarray Analysis [75,76] was implemented to perform background correction. Intensities for three biological replicates were summarized with quantile normalization [77]. In addition, intensities for the three biological replicates of *As *and *F*_*1*_*As *were summarized altogether with quantile normalization. Consistency and density plots may be found in the Additional files. PM probes exhibited some mismatches for the *At *genotype, as this array is based on a different reference; the arrays exhibited an additional lower hybridization intensity peak. PM probes from conserved exon regions were much more robust.

As expected from interspecific sequence divergence, the number of *Aa *higher intensity probes decreased, while the number of lower intensity probes increased. Note, however, that 'conservative features' and 'divergent features' peak at similar intensities in both species, making the analyses easier. Similar to *At*, *Aa *lower intensity probes were overrepresented in non-coding regions.

### Identifying *As *genomic regions with putative multi-gene alterations

Probe intensities among three biological replicates in *As *were averaged and paired with the corresponding average among the three *F*_*1*_*As *replicates. For each gene, a paired Wilcoxon rank-sum test (FDR <0.05) [78] of all probes was used to identify genes with differential hybridization. The significance of individual genes might be misleading, but the pattern for multigene regions is robust. We scanned for windows in which at least 27 (90%) out of 30 genes exhibited unidirectional stronger or unidirectional weaker hybridization in *As *in comparison with *F*_*1*_*As*. We also required these differences to be significant at FDR <0.05 for at least 9 (30%) genes. Overlapping windows were collapsed to identify the entirety of these regions.

### Multi-genotype array normalization and identification of diagnostic features

Our goal here is to select probe features enabling the comparison of *At *and *Aa *signal representation in *As *DNA and RNA. To enable cross-comparison of DNA and RNA, the analyses have to be made gene-by-gene, with DNA and RNA hybridization signals normalized to the same level with each gene.

First, probes representing conserved signatures between genotypes were identified and used to scale the entire gene. For every probe in a gene, its average intensity among replicates in *At *was compared to the average intensity in *Aa*. These ratios formed a unimodal distribution and the peak of this distribution was used as the scaling factor for which to normalize between genotypes for that gene. Mathematically, for probe *i *in the gene, the average intensity among *j *biological replicates in both genotypes is defined as:

$$A_{i}=\frac{1}{3}\sum_{j=1}^{3}a_{ij}\text{ and }T_{i}=\frac{1}{3}\sum_{j=1}^{3}t_{ij}$$

where *a*_*ij *_and *t*_*ij *_represent the probe intensities of the *j*th replicate of the *i*th probe in *Aa *and *At*, respectively. Defining *X*_*i *_as:

$$X_{i}=\frac{T_{i}}{A_{i}}\sim f(x)$$

The scaling factor, *x*_*max *_is defined as:

$$x_{\max}=\underset{x}{\arg\max}f(x)$$

The value for *x*_*max *_was estimated using the mlv function in R, which calculates the kernel density and searches for *x *that maximizes that estimated density function. From hereon, we replace all *a*_*ij *_values with rescaled values represented by product(*x*_*max*_,*a*_*ij*_). We disregarded genes whose *f(x) *failed the Shapiro-Wilks normality test. This normalization method is similar to one recently outlined by Robinson and Oshlack [79], where a scaling parameter is used to normalize between two samples.

Second, we identified single feature polymorphisms or DFs between *At *and *Aa *using a Welch *t*-test of log2-transformed values, followed by controlling FDR to be smaller than 0.05. These approaches enabled us to analyze homoeolog-specific retention in 24,344 out of approximately 39,000 *At *genes.

### Analysis of DFs in DNA samples from *As*

If an *As *gene retained both parental homoeologs, we should observe an equal mix of *At *and *Aa *signals. A linear model was used to determine whether *As *has probe intensities within a gene contributed by i) both parents (mixed), ii) parental *At *only (*At*-like), or iii) parental *Aa *only (*Aa*-like). For a gene with n DFs, the vector of intensities, S = [S_1_, S_2_,..., S_n_], may be contributed by corresponding *Aa*- and *At*-specific signals, such that S = α1•A + β1•T and the contribution of *Aa*, α1, can be estimated using a simple linear regression. Specifically:

$$S_{ij}=\alpha_{1}A_{i}+\beta_{1}T_{i}+\varepsilon_{ij}$$

where *i *= 1,2,..,n, *j *= 1,2,3 for the three biological replicates, and *A*_*i *_and *T*_*i *_are the mean intensities in *Aa *and *At*, respectively. *ε*_*ij *_are error terms that are independent random variables from a normal distribution with a mean 0 and variance σ^2^. The strength of our experimental design is in *F*_*1*_*As*, in which a null model holds true for genomic DNA. For *F*_*1*_*As*, this expectation is:

$$F_{ij}=\alpha_{2}A_{i}+\beta_{2}T_{i}+\varepsilon_{ij}$$

To detect deviations from the null, we tested whether α1 is significantly different from α2. Under the null hypothesis that α1 = α2, and assuming α + β = 1:

$$X=\frac{\frac{1}{2}{\left({\overset{\wedge }{\alpha }}_{1}-{\overset{\wedge }{\alpha }}_{2}\right)}^{2}\sum_{i=1}^{3n}{\left(A_{i}-T_{i}\right)}^{2}}{\left[\frac{\sum_{i=1}^{3n}\left({\left(S_{i}-T_{i}-{\overset{\wedge }{\alpha }}_{1}(A_{i}-T_{i})\right)}^{2}+{\left(F_{i}-T_{i}-{\overset{\wedge }{\alpha }}_{2}(A_{i}-T_{i})\right)}^{2}\right)}{6n-1}\right]}$$

follows an F distribution with 1 and 6n - 1 degrees of freedom. This assumption of α + β = 1 can be made since the contributions of *Aa *and *At *are weighted. The bias was labeled as *Aa*-like if α1 > α2 and as *At*-like if α1 < α2. To account for multiple testing issues arising from thousands of genes tested, Benjamini-Hochberg's FDR was employed to adjust the significance level at 0.05 [78].

As with all linear regression models, we assume that the error terms follow a normal distribution. We investigated this by applying a Shapiro-Wilks test on each gene to ensure that they were normal. We removed over 7,000 genes that failed these tests. We found little discrepancy for the results of the analyses when α1 was defined as the *At *contribution. We also determined significance by performing a permutation test for each gene and found little discrepancy with the F distribution shown above.

### Analysis of DFs in *As *transcripts

Since we are estimating the relative contribution of *Aa *rather than the absolute, the expression level of every gene in the *As *transcriptome was normalized to identical hybridization levels with its corresponding genomic DNA. This was done using probes representing conserved signatures, identified as previously described. We then analyzed the homoeolog-specific expression with the same linear model approach as above, using DFs identified between RNA and DNA, and α found in *As *DNA as the null reference point. When these intensities of DFs are biased in one direction, we can determine homoeolog-specific expression. Furthermore, for each gene, α was estimated by regressing over all DFs in the set, minimizing spurious effects of individual probes. Forty-nine percent of genes were expressed. Distributions of intensities for conserved features in *As *DNA and RNA prior to and after gene-wise normalization are shown in the Additional files. The homoeolog-specific expression was assayed in 18,876 genes.

### Illumina data analysis

Pair-ended 72-base Illumina reads were aligned and mapped allowing up to 10 mismatches using bwa [80] to 102 *Aa *transcript sequences and their orthologous *At *sequences. A pairwise global alignment identified SNPs and short insertion/deletion variants between orthologous *Aa *and *At *gene pairs. Reads that mapped to either of the two orthologs were scanned for these variants to ensure that they were clustered with the appropriate ortholog (Figure 6). The number of reads mapped to each ortholog was normalized to FPK (fragments per kilobase of exon) to account for slightly variable sequence length between orthologs. This analysis and its results are summarized in Figures 6 and 7, and in Additional file 5.

### Variation within *Aa *and *At*

**Figure 9:**
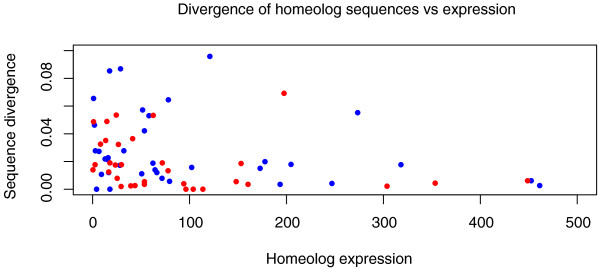
**Divergence of *At *and *Aa *homoeologs in *As *in comparison with *At *and *Aa *references (Y-axis) compared to homoeolog-specific expression (X-axis)**.

Note that although extant accessions of *Aa*, *At*, and *F*_*1*_*As *were used, *As *was formed 12 to 300 KYA, perhaps from different accessions. DFs and Illumina resequencing may potentially result in misleading conclusions. Nevertheless, 5 million years of sequence divergence between *Aa *and *At *compares favorably with the smaller amount of standing sequence variation and with the unaccounted extra divergence since *As *formation. From the above resequencing data, we estimated the divergence of the *Aa *homoeolog within *As *from the homologous gene in *Aa*. Likewise, we estimated the divergence of the *At *homoeolog within *As *from the homologous gene in *At*. Consistent with high sequence variation in *Aa *[43], the divergence from parental homologs is larger in *Aa*, as sequence variation in natural *At *is very limited [42]. This would result in fewer *Aa*-like calls, and lower biases detected in this manuscript. Note that, as expected from [66], stronger expressed genes appear more conserved and exhibit lesser *Aa *and *At *divergences (Figure 9).

## Abbreviations

*Aa: Arabidopsis arenosa*; AFLP: amplified fragment length polymorphism; *As: Arabidopsis suecica*; *At: Arabidopsis thaliana*; DF: diagnostic feature; *F*_*1*_*As*: F_1_ artificial allotetraploid; FDR: false discovery rate; KYA: thousand years ago; SNP: single-nucleotide polymorphism.

## Competing interests

The authors declare that they have no competing interests.

## Authors' contributions

PLC performed the computational and statistical analysis of the data, carried out the molecular resequencing, and drafted the manuscript. BPD performed sequence extraction and microarray experiments, and drafted the manuscript. MM performed sequence extraction and microarray experiments. LC participated in the design of the study. SVN conceived the study, participated in its design and coordination, and drafted the manuscript. All authors read and approved the final manuscript.

## Supplementary Material

Additional file 1 **Differential hybridization between *As *and *F***_***1***_***As***. Excel table showing 6,790 genes with differential hybridization between *As *and *F*_*1*_*As *(Wilcoxon ranked sum test, FDR <0.05). Click here for file

Additional file 2 **Differentially hybridized clusters between *As *and *F***_***1***_***As***. Excel table showing 1,643 genes found within differentially hybridized clusters between *As *and *F*_*1*_*As*. Clusters contain at least 30 genes with a strong unidirectional bias, where at least 27 genes have the same bias, and significant for at least 9 genes. Click here for file

Additional file 3 **Outline of genes included and analyzed**. Excel table outlining the number of genes discarded and included at each step in the analysis. Click here for file

Additional file 4 **Homoeolog-specific retention in *As *DNA**. Excel table showing 938 genes with homoeolog-specific retention in *As *DNA. Click here for file

Additional file 5 **Comparison of homoeolog-specific expression estimated from *At *tiling microarray and Illumina resequencing**. Excel table showing comparison of expression for 102 genes using both *At *tiling microarrays and Illumina resequencing. Click here for file

Additional file 6 **Summary of probe hybridization intensities between *At*, *Aa*, *As*, and *F***_***1***_***As. ***Probe hybridization intensities are shown for various regions throughout the genome (Figures S1 to S12). Density plots are shown for probe hybridization of DNA for PM and MM probes (Figures S13 to S16). A density plot is shown for conserved probes in *As *DNA and *As *RNA before and after gene-level normalization. Click here for file
