# Supplementary material for: Homoeolog-specific retention and use in allotetraploid Arabidopsis suecica depends on parent of origin and network partners
Source: Genome Biol. 2010 Dec 23;11(12):R125. doi: 10.1186/gb-2010-11-12-r125 (PMC3046485; doi:10.1186/gb-2010-11-12-r125)
Supplement: Additional file 6 — Summary of probe hybridization intensities between At, Aa, As, and F1As. Probe hybridization intensities are shown for various regions throughout the genome (Figures S1 to S12). Density plots are shown for probe hybridization of DNA for PM and MM probes (Figures S13 to S16). A density plot is shown for conserved probes in As DNA and As RNA before and after gene-level normalization. [file gb-2010-11-12-r125-S6.pdf]

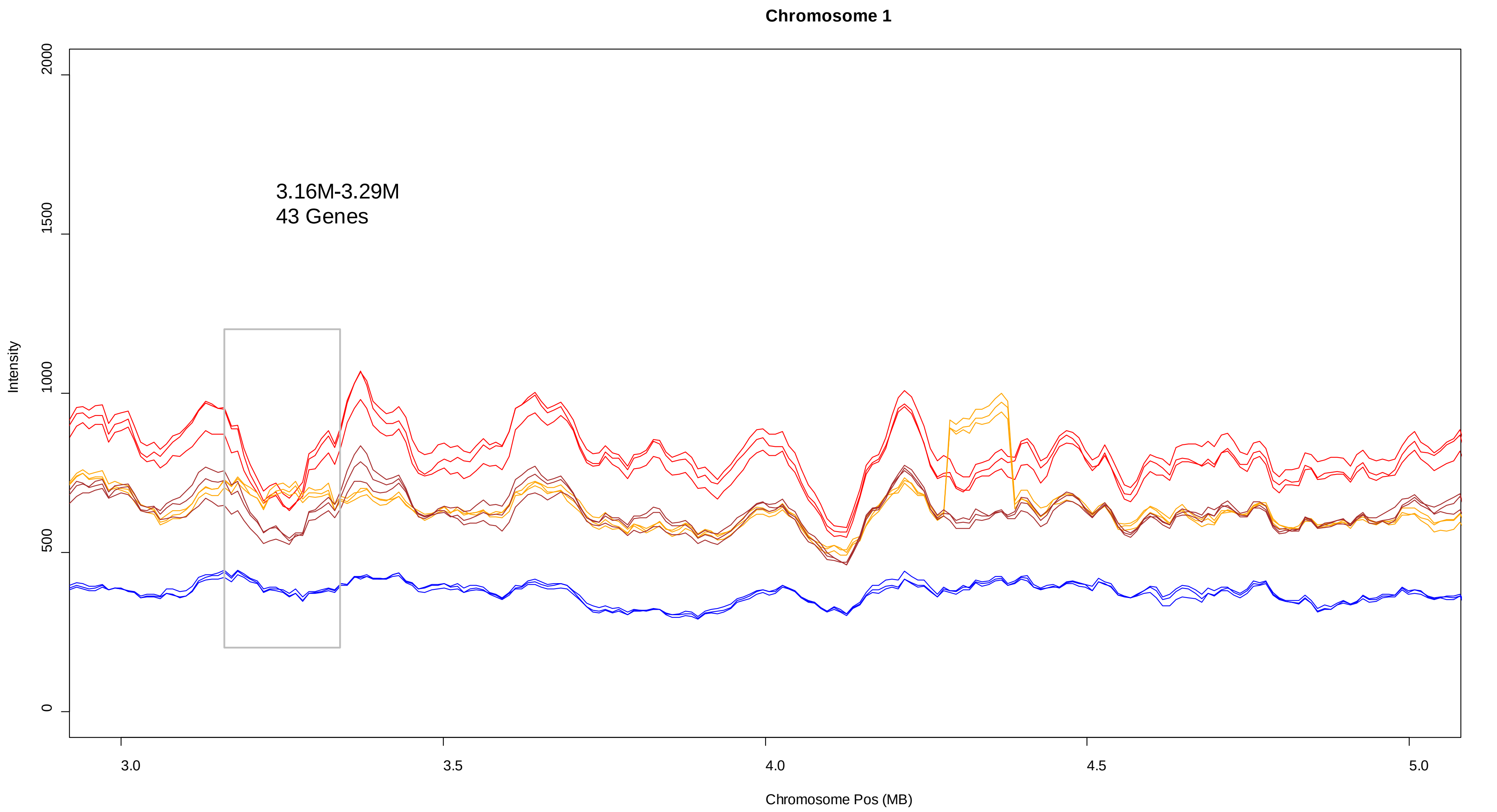

Figure S1: **Chromosomal distribution of probe intensities**. 100kb sliding window averages for ***At*** (red), ***Aa*** (blue), ***As*** (gold), and ***F_1As*** (brown). See genes from these regions in Additional File 2.

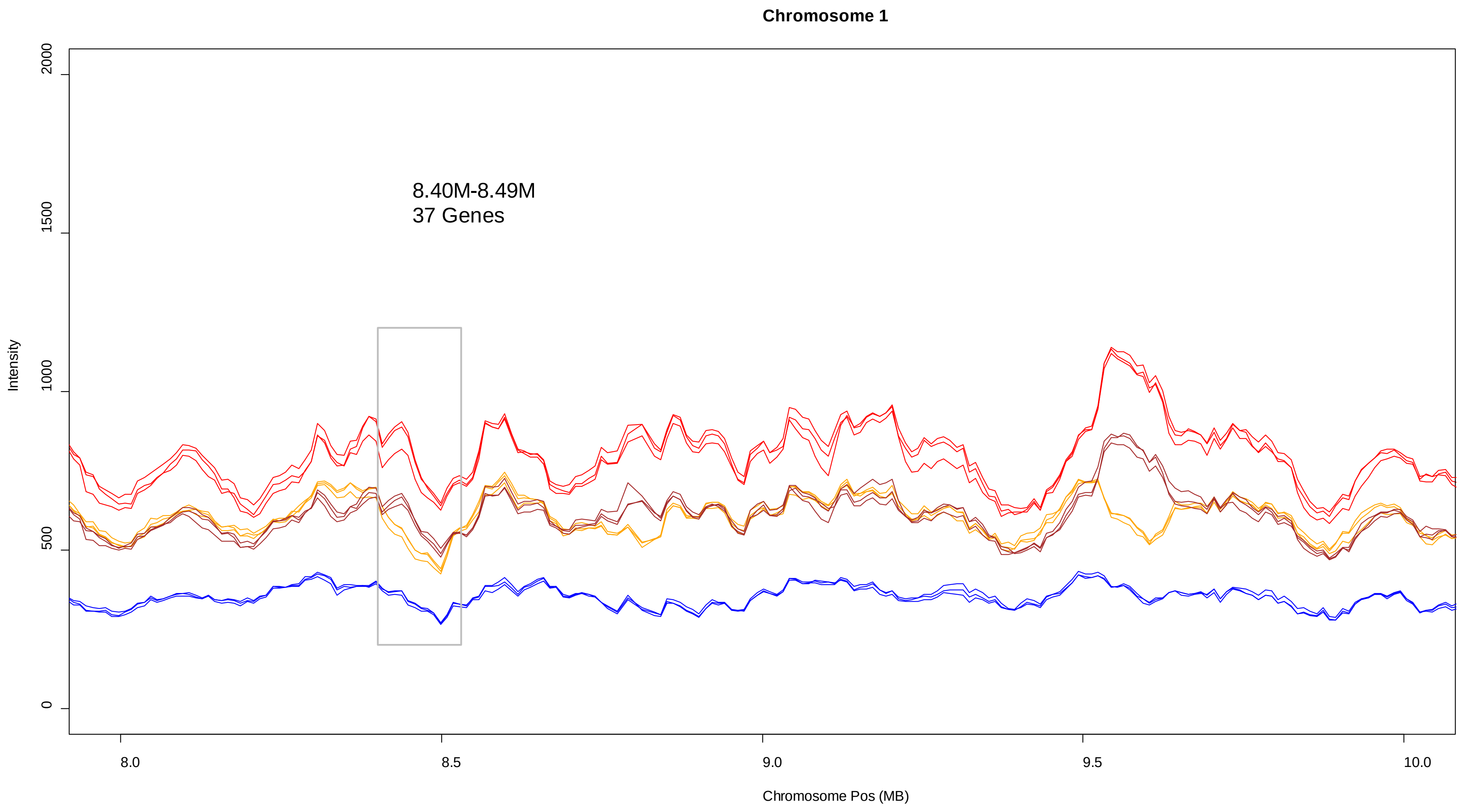

Figure S2: **Chromosomal distribution of probe intensities**. 100kb sliding window averages for ***At*** (red), ***Aa*** (blue), ***As*** (gold), and ***F_1As*** (brown). See genes from these regions in Additional File 2.

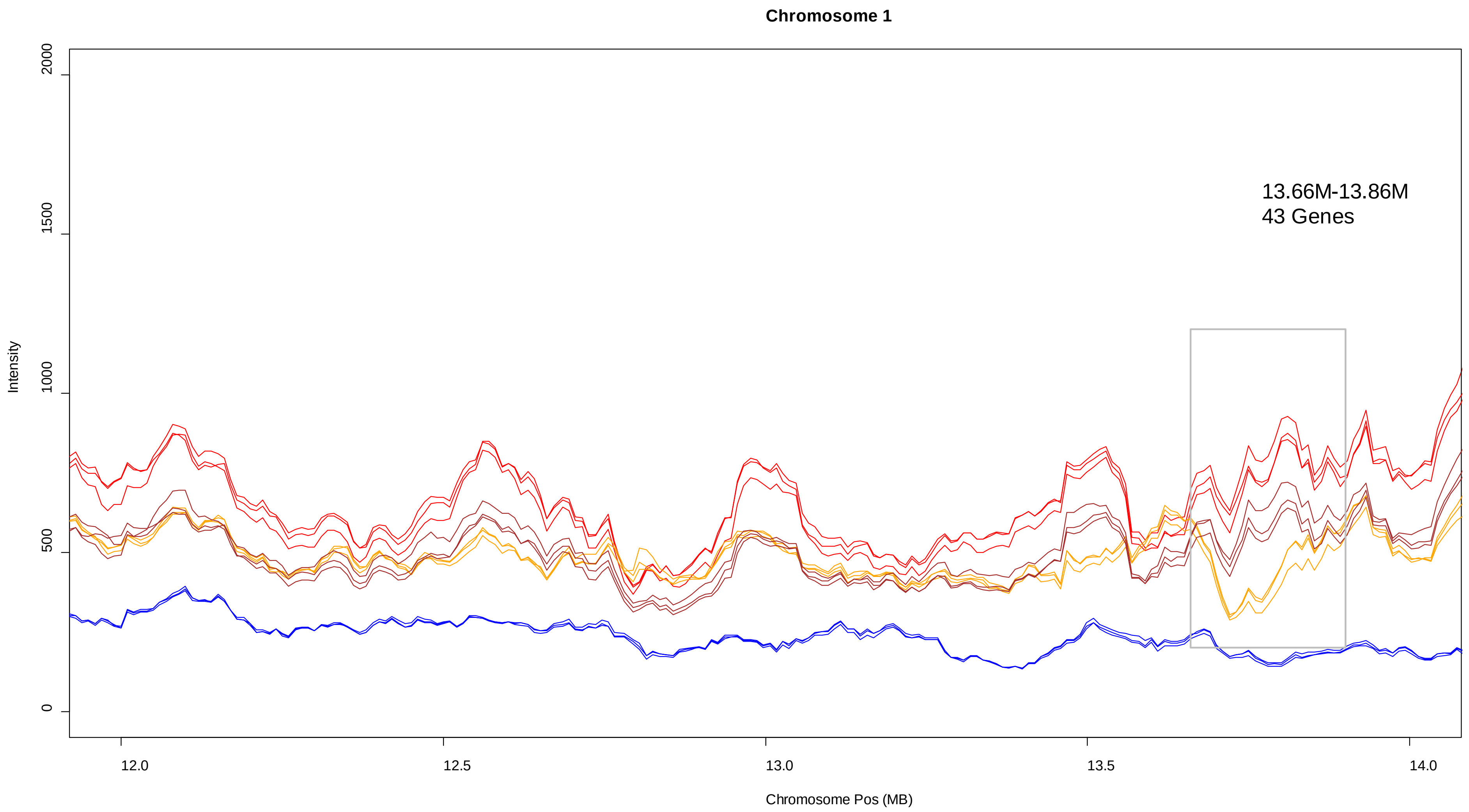

Figure S3: **Chromosomal distribution of probe intensities**. 100kb sliding window averages for ***At*** (red), ***Aa*** (blue), ***As*** (gold), and ***F_1As*** (brown). See genes from these regions in Additional File 2.

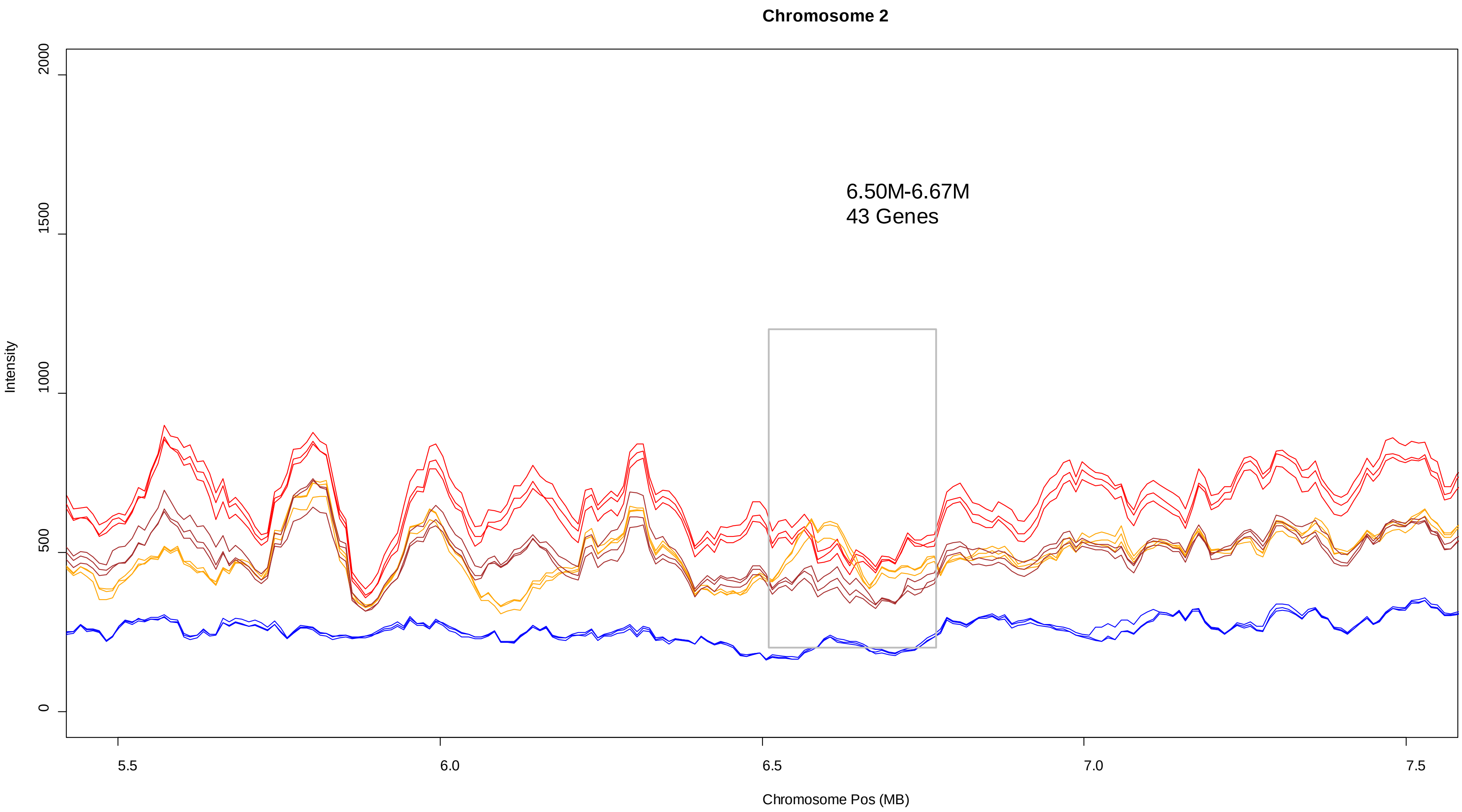

Figure S4: **Chromosomal distribution of probe intensities**. 100kb sliding window averages for ***At*** (red), ***Aa*** (blue), ***As*** (gold), and ***F_1As*** (brown). See genes from these regions in Additional File 2.

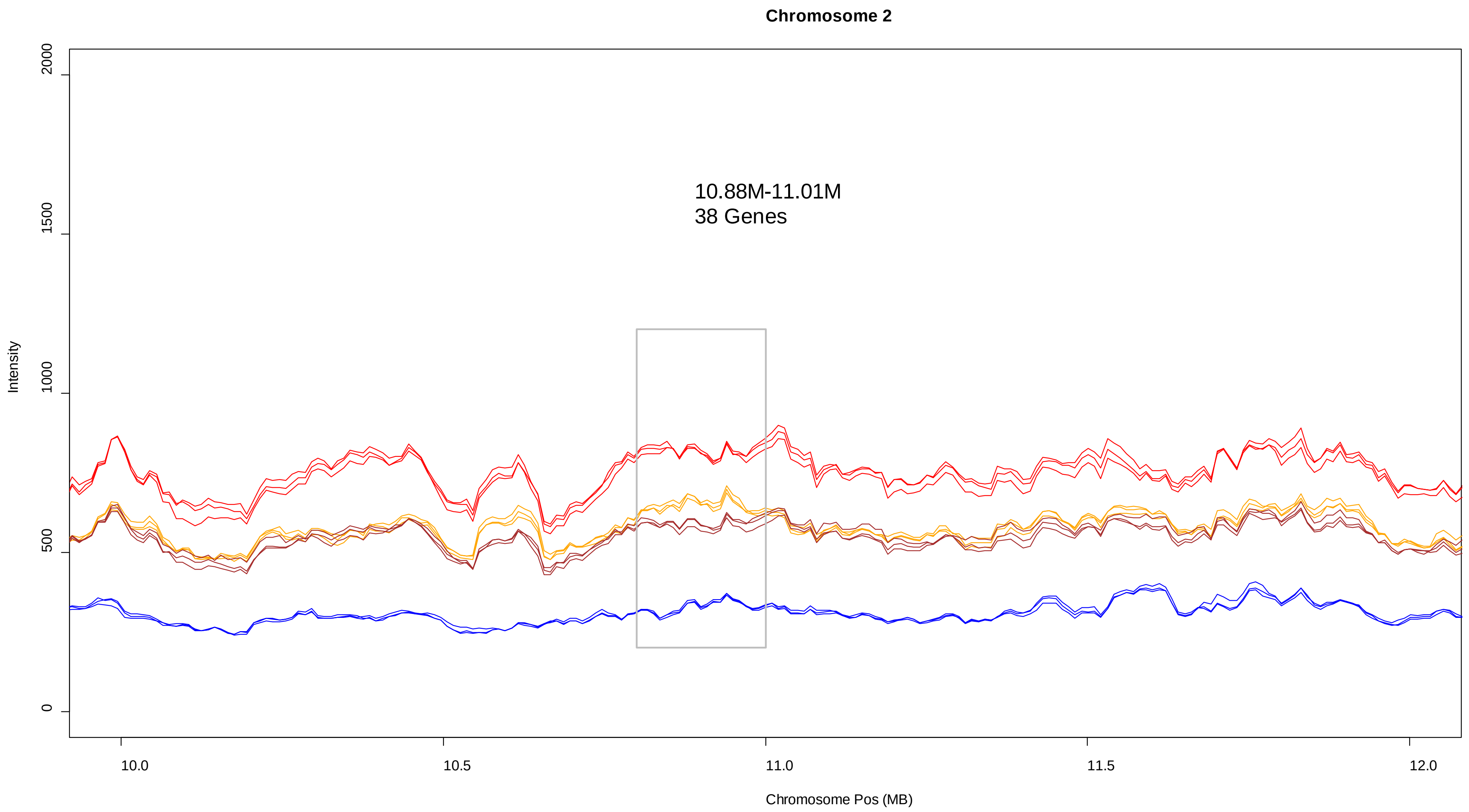

Figure S5: **Chromosomal distribution of probe intensities**. 100kb sliding window averages for ***At*** (red), ***Aa*** (blue), ***As*** (gold), and ***F_1As*** (brown). See genes from these regions in Additional File 2.

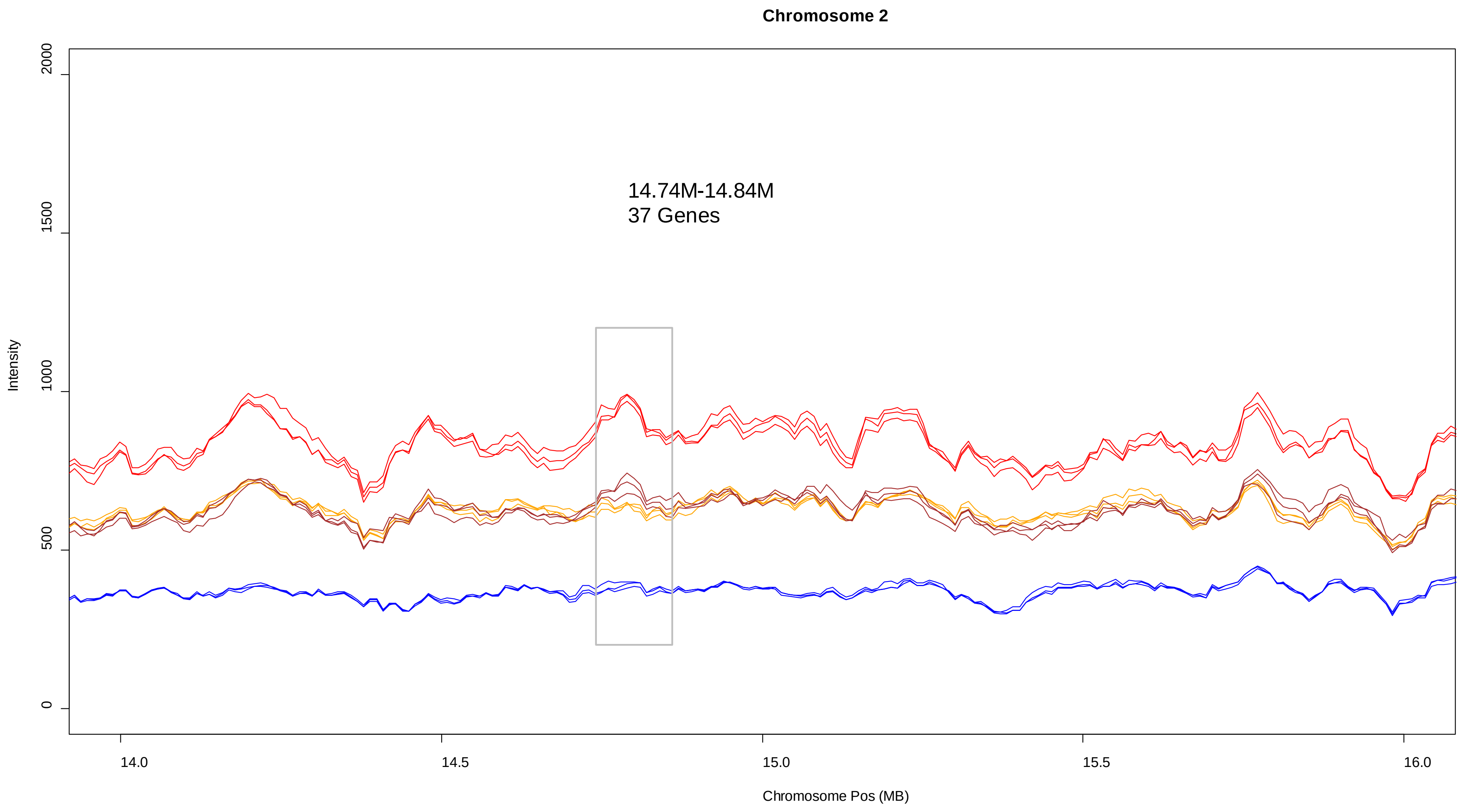

Figure S6: **Chromosomal distribution of probe intensities**. 100kb sliding window averages for ***At*** (red), ***Aa*** (blue), ***As*** (gold), and ***F_1As*** (brown). See genes from these regions in Additional File 2.

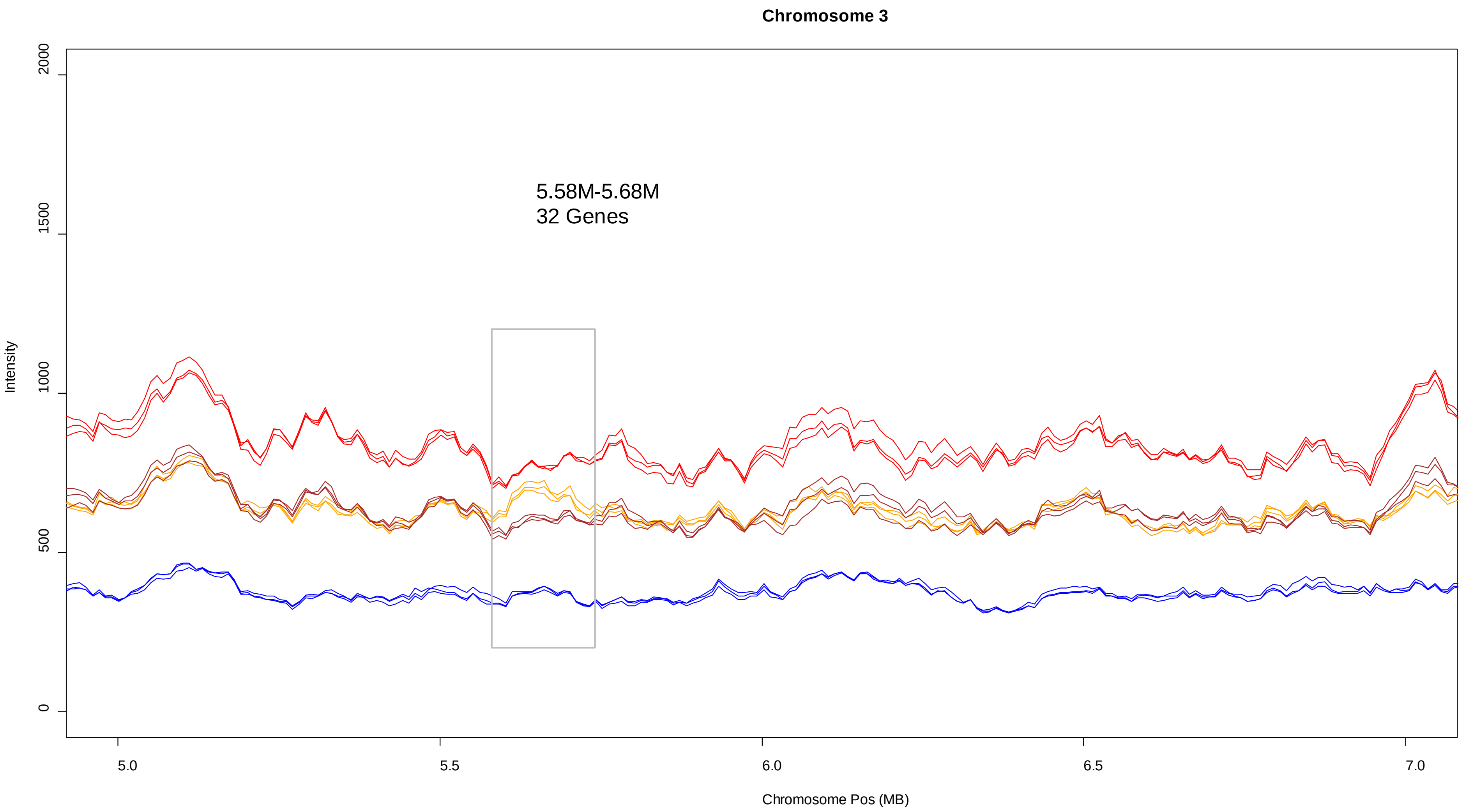

Figure S7: **Chromosomal distribution of probe intensities**. 100kb sliding window averages for ***At*** (red), ***Aa*** (blue), ***As*** (gold), and ***F_1As*** (brown). See genes from these regions in Additional File 2.

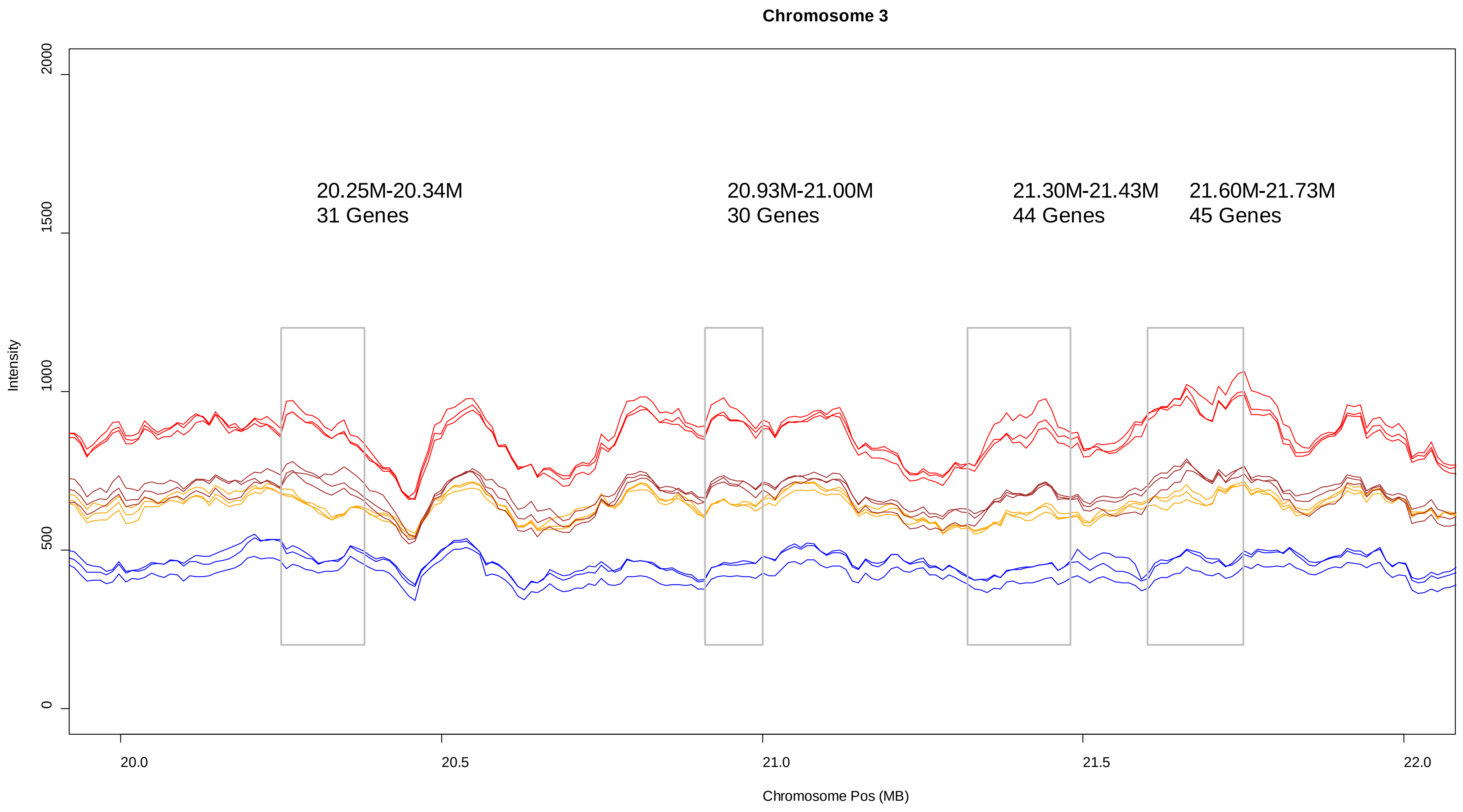

Figure S8: **Chromosomal distribution of probe intensities**. 100kb sliding window averages for ***At*** (red), ***Aa*** (blue), ***As*** (gold), and ***F_1As*** (brown). See genes from these regions in Additional File 2.

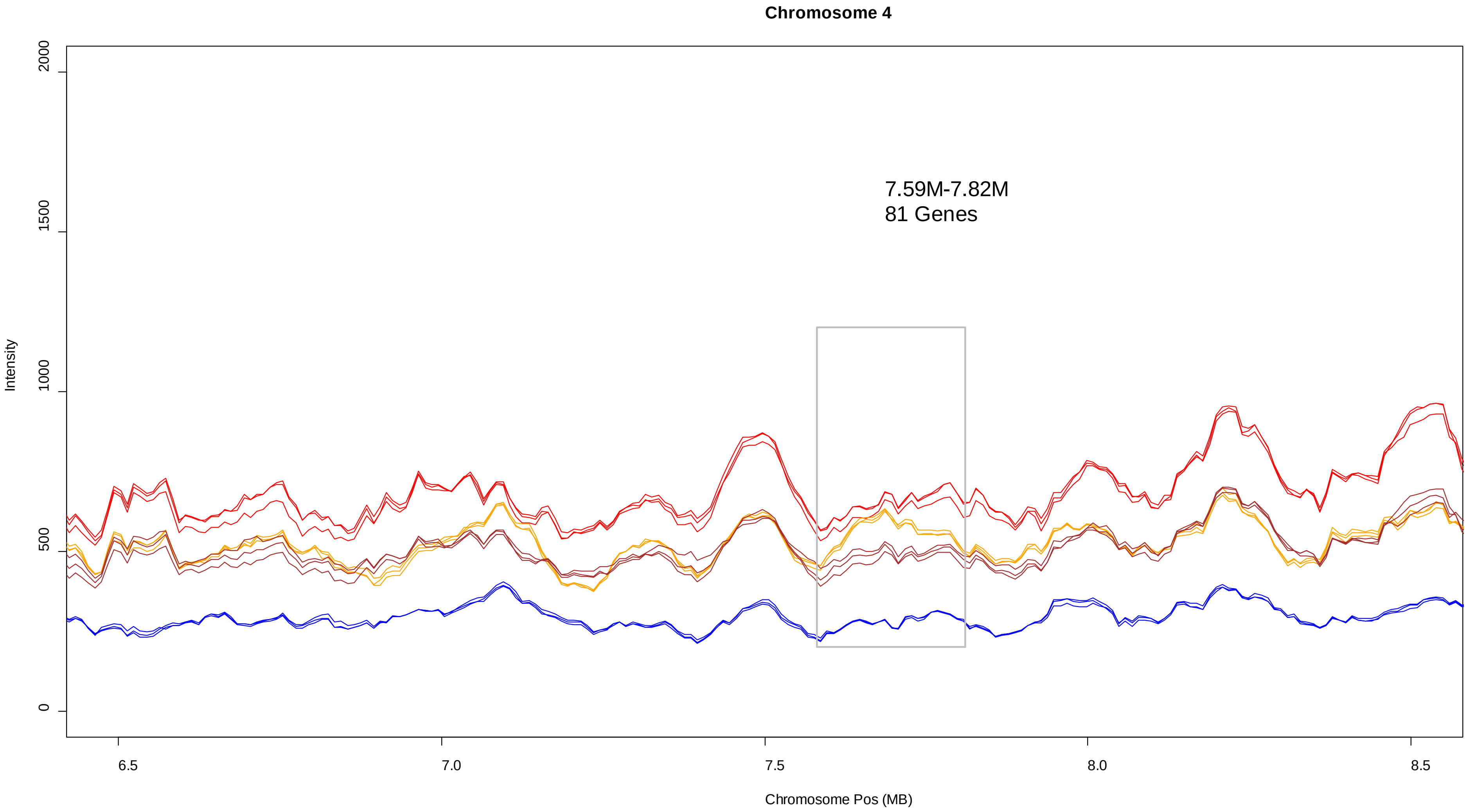

Figure S9: **Chromosomal distribution of probe intensities**. 100kb sliding window averages for ***At*** (red), ***Aa*** (blue), ***As*** (gold), and ***F_1As*** (brown). See genes from these regions in Additional File 2.

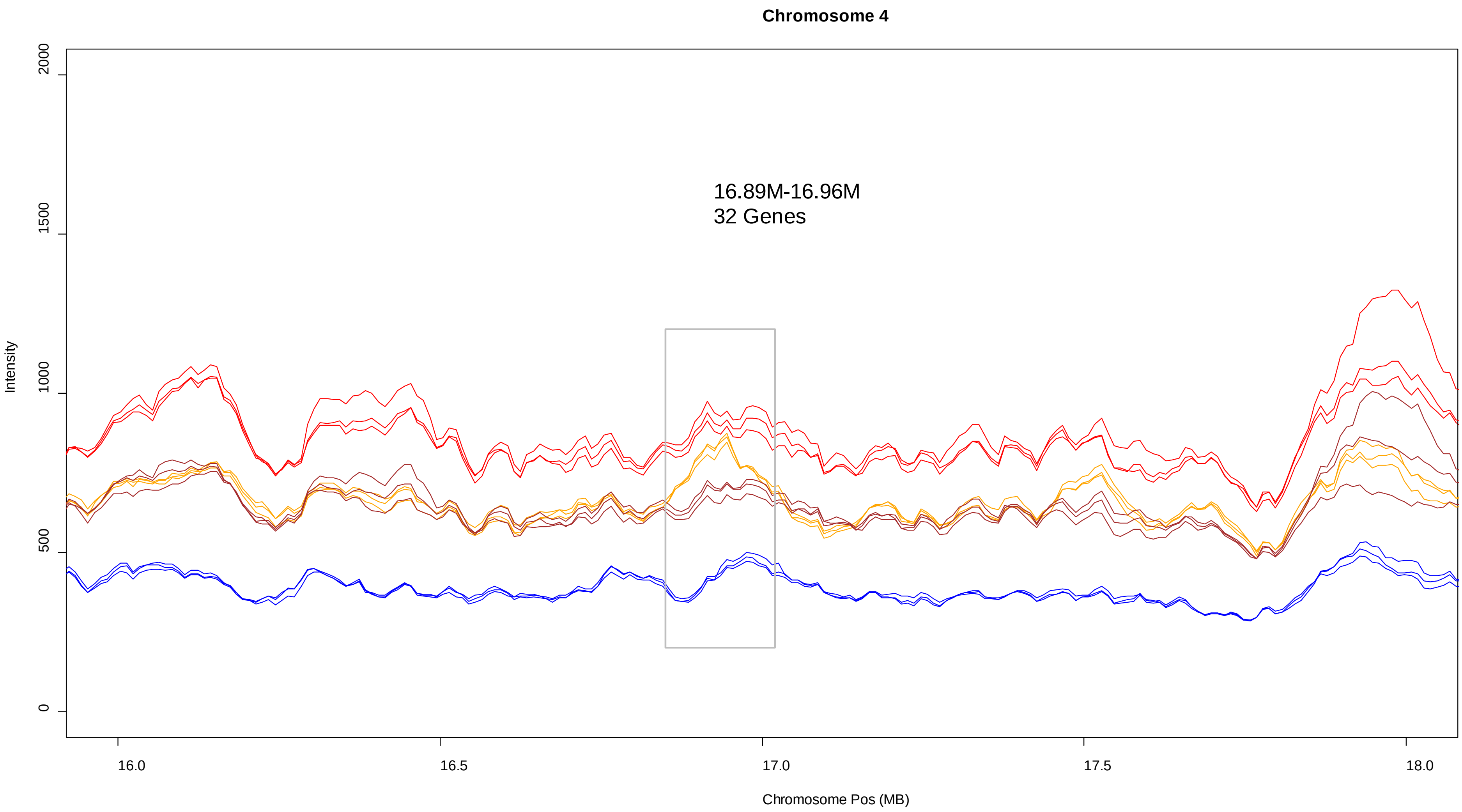

Figure S10: **Chromosomal distribution of probe intensities**. 100kb sliding window averages for ***At*** (red), ***Aa*** (blue), ***As*** (gold), and ***F_1As*** (brown). See genes from these regions in Additional File 2.

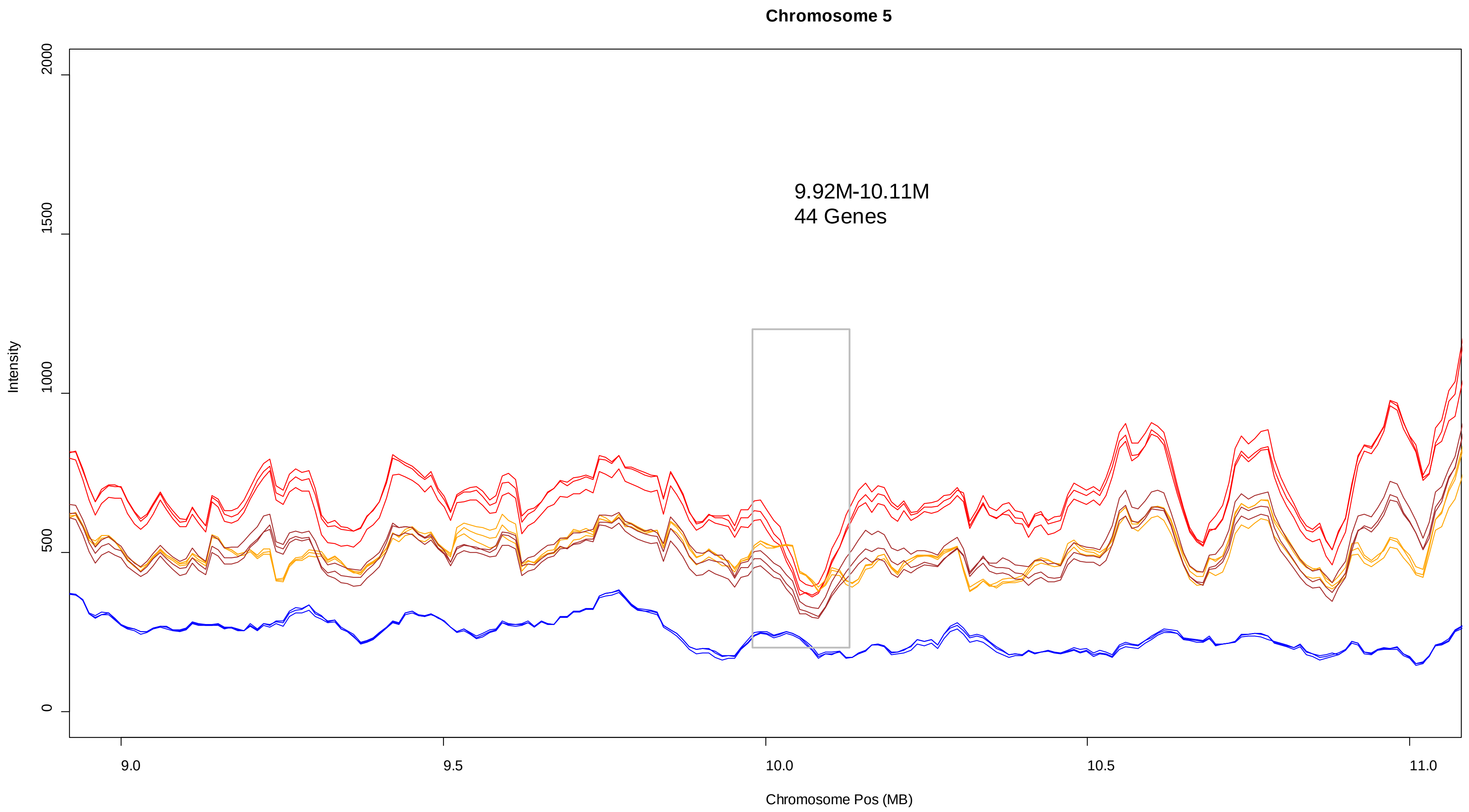

Figure S11: **Chromosomal distribution of probe intensities**. 100kb sliding window averages for ***At*** (red), ***Aa*** (blue), ***As*** (gold), and ***F_1As*** (brown). See genes from these regions in Additional File 2.

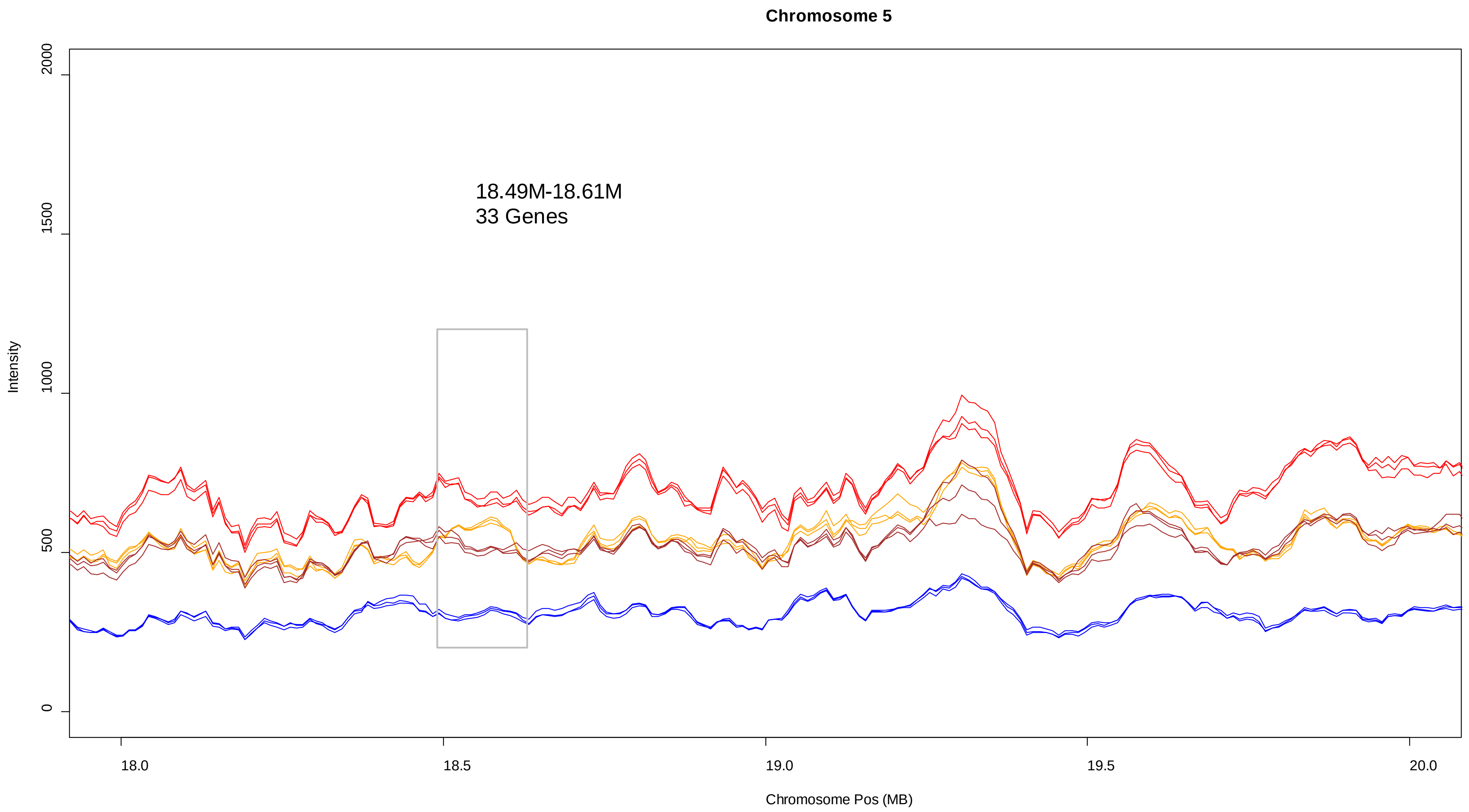

Figure S12: **Chromosomal distribution of probe intensities**. 100kb sliding window averages for ***At*** (red), ***Aa*** (blue), ***As*** (gold), and ***F_1As*** (brown). See genes from these regions in Additional File 2.

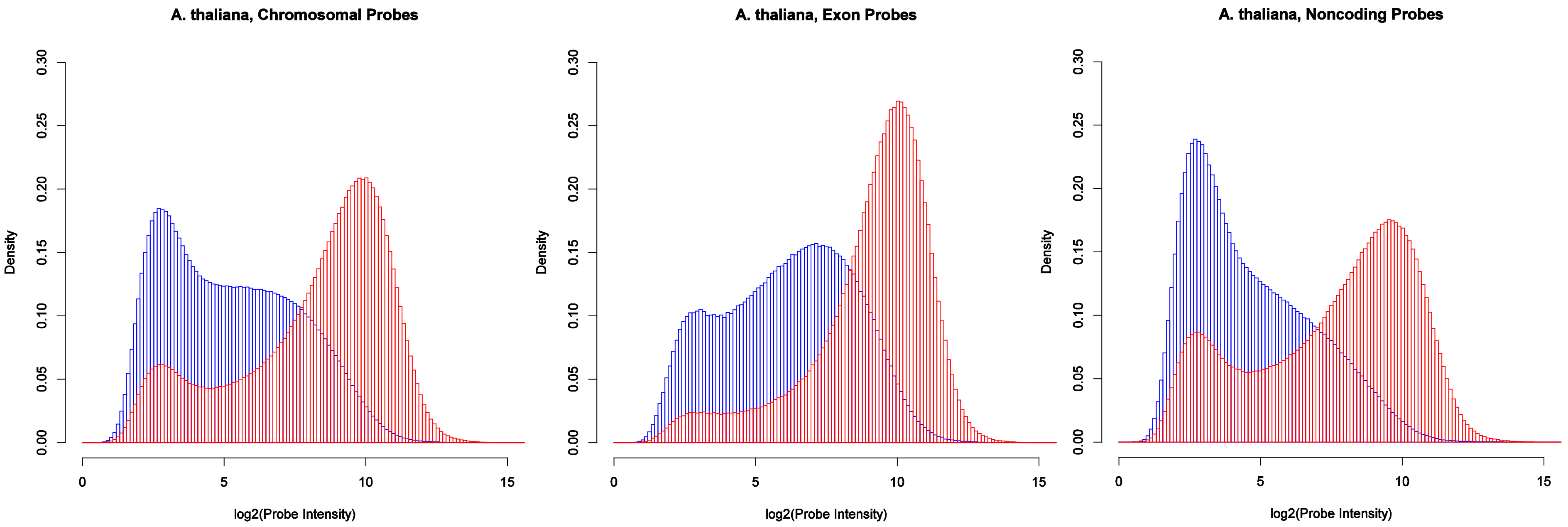

Figure S13: Distribution of Probe Intensities in ***At*** for Chromosomal, Exon, and Noncoding Probes (L to R). PM intensities are denoted in red. MM intensities are denoted in blue.

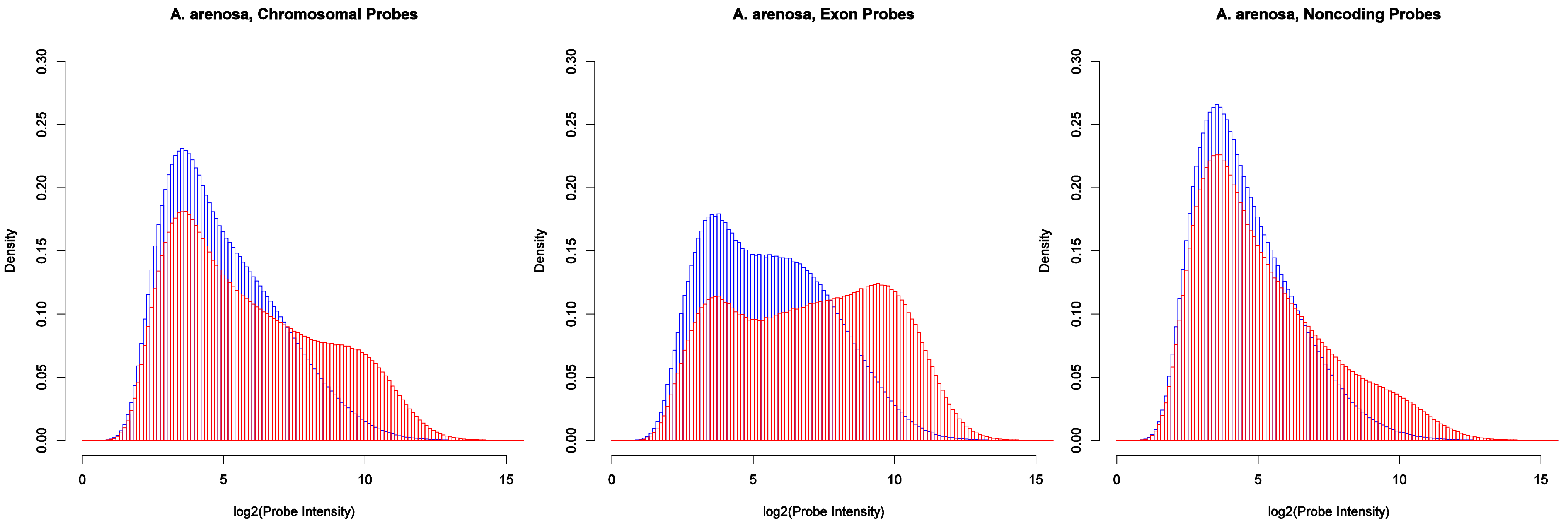

Figure S14: Distribution of Probe Intensities in ***Aa*** for Chromosomal, Exon, and Noncoding Probes (L to R). PM intensities are denoted in red. MM intensities are denoted in blue.

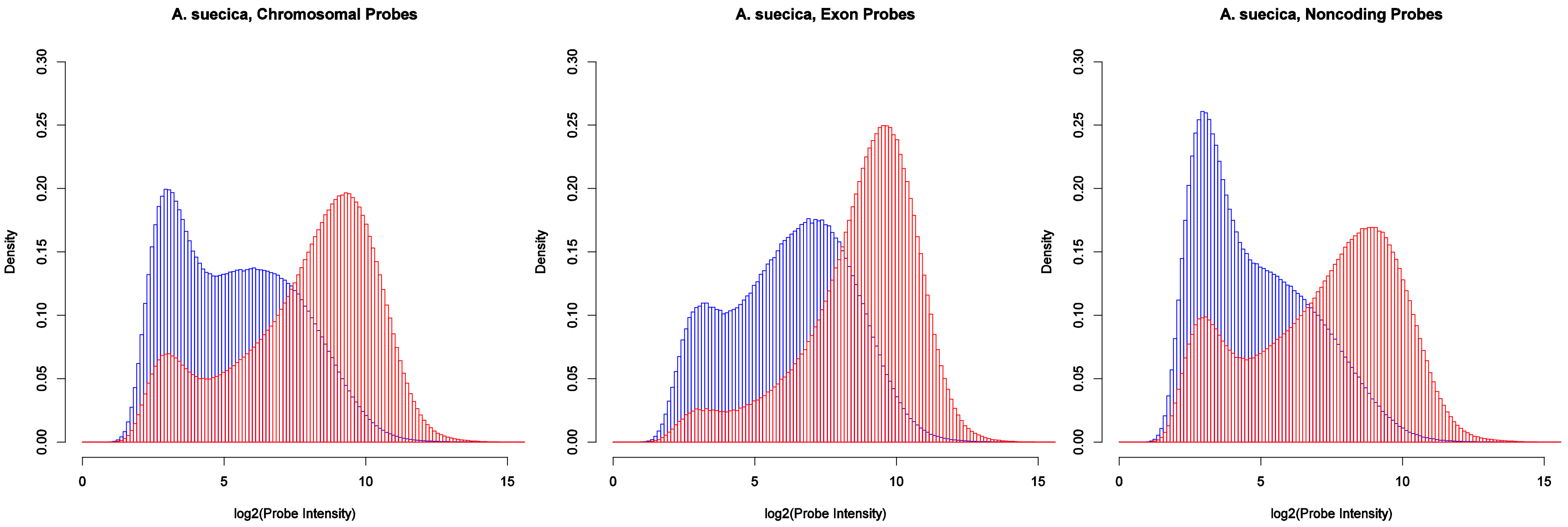

Figure S15: Distribution of Probe Intensities in ***As*** for Chromosomal, Exon, and Noncoding Probes (L to R). PM intensities are denoted in red. MM intensities are denoted in blue.

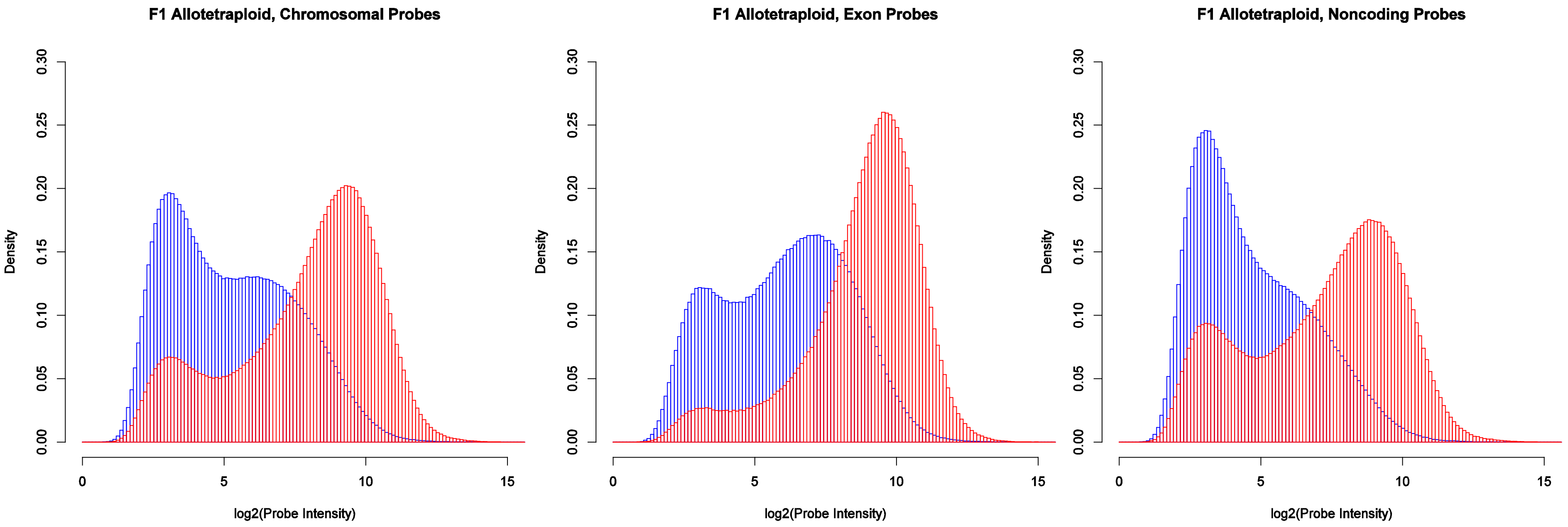

Figure S16: Distribution of Probe Intensities in $\boldsymbol{F_1As}$ for Chromosomal, Exon, and Noncoding Probes (L to R). PM intensities are denoted in red. MM intensities are denoted in blue.

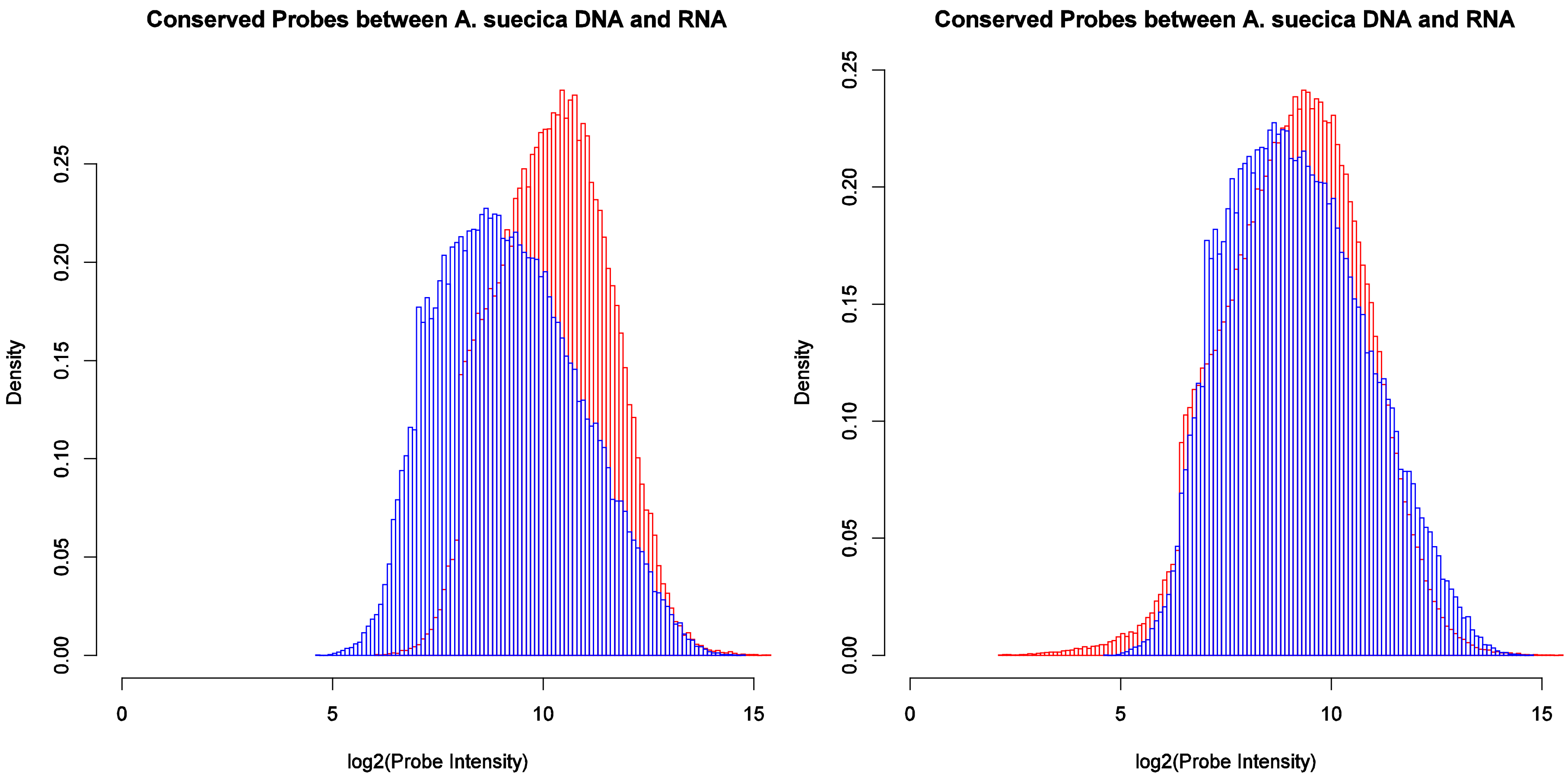

Figure S17: Distribution of Conserved Probe Intensities in ***As*** DNA (blue) and ***As*** RNA (red) before (L) and after (R) gene-level normalization.
